# A heterotrimeric protein complex assembles the metazoan V-ATPase upon dissipation of proton gradients

**DOI:** 10.1038/s41594-025-01610-9

**Published:** 2025-07-11

**Authors:** Christopher Nardone, Julian Mintseris, Dingwei He, Justine C. Rutter, Benjamin L. Ebert, Steven P. Gygi, Tom Rapoport

**Affiliations:** 1 Department of Cell Biology, Harvard Medical School, Boston, MA USA; 2 Howard Hughes Medical Institute, Boston, MA USA; 3 Department of Medical Oncology, Dana-Farber Cancer Institute, Boston, MA USA; 4 Cancer Program, Broad Institute of MIT and Harvard, Cambridge, MA USA

**Keywords:** Lysosomes, Molecular biology, Membrane proteins

## Abstract

Organelles such as lysosomes and synaptic vesicles are acidified by V-ATPases, which consist of a cytosolically oriented V_1_ complex that hydrolyzes ATP and a membrane-embedded V_O_ complex that pumps protons. In yeast, V_1_–V_O_ association is facilitated by the RAVE (regulator of H^+^-ATPase of the vacuolar and endosomal membrane) complex, but how higher eukaryotes assemble V-ATPases remains unclear. Here we identify a metazoan RAVE complex (mRAVE) whose structure and composition are notably divergent from the ancestral counterpart. mRAVE consists of DMXL1 or DMXL2, WDR7 and the central linker ROGDI. DMXL1 and DMXL2 interact with subunits A and D of the inactive, isolated V_1_. On dissipation of proton gradients, mRAVE binds to V_1_ and V_O_, forming a supercomplex on the membrane. mRAVE then catalyzes V_1_–V_O_ assembly, enabling lysosomal acidification, neurotransmitter loading into vesicles and ATG16L1 recruitment for LC3/ATG8 conjugation onto single membranes. Our findings provide a molecular basis for neurological disorders caused by mRAVE mutations.

## Abstract (web summary)

Nardone et al. identify a metazoan protein complex (mRAVE) that assembles V-ATPases from two subcomplexes, enabling lysosome acidification, neurotransmitter vesicle loading and a lysosome damage response pathway.

## Main

The vacuolar adenosine triphosphatase (V-ATPase) is a large, membrane-embedded proton pump that acidifies endosomes, lysosomes and the *trans*-Golgi network in all eukaryotic cells^1^. It regulates glycosylation in the Golgi, the reversible binding of cargo receptors in vesicular transport and the degradation of cellular material in lysosomes^2^. In specialized cell types, the V-ATPase is localized to the plasma membrane and acidifies the extracellular environment, which is crucial for bone remodeling by osteoclasts and acid–base homeostasis in distal segments of the kidney tubule^3^. In neurons, the V-ATPase is required to package certain neurotransmitters into synaptic vesicles^4,5^. Misregulation of the V-ATPase causes osteopetrosis, renal tubular acidosis, neurodevelopmental and neurodegenerative diseases. Cancer cells also deploy V-ATPases to acidify the tumor microenvironment, promoting cell invasion and metastasis^6,7^.

**Fig. 1:**
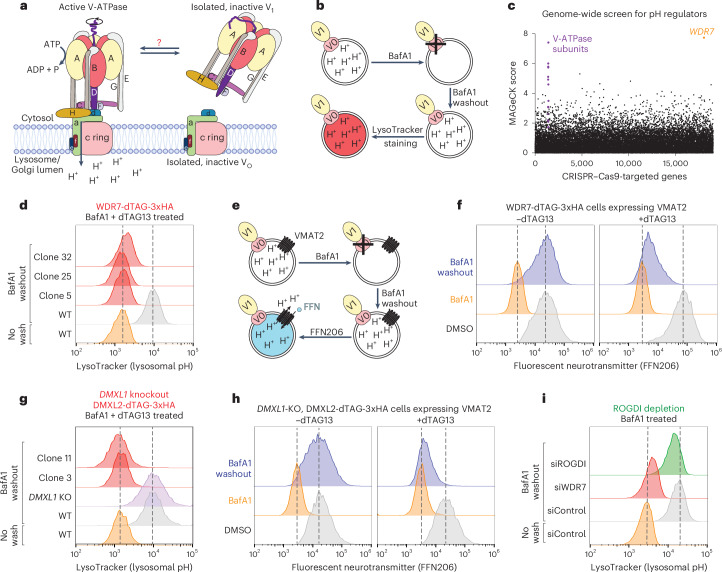
mRAVE is required for lysosomal acidification and neurotransmitter loading into vesicles. **a**, Schematic of the assembled V-ATPase and its reversible dissociation into the V_1_ and V_O_ subcomplexes. **b**, Schematic of the assay to measure reacidification of lysosomes after BafA1 treatment. **c**, HEK-293T cells stably expressing a genome-wide CRISPR–Cas9 library were treated with BafA1. BafA1 was removed and the cells were recovered in fresh medium. Cells were then stained with LysoTracker and subjected to cell sorting, followed by sequencing of the guide RNAs enriched in the lowest 5% fluorescent population relative to the input. Each dot shows a gene with the associated enrichment score based on MAGeCK^67^. **d**, WT HEK-293T cells or different clones of edited cells expressing WDR7-dTAG-3xHA from the endogenous locus were pretreated with the degrader dTAG13. Then, a similar assay to **c** was followed, keeping dTAG13 present throughout. Fluorescence was measured by flow cytometry. **e**, Schematic of the assay to measure neurotransmitter (FFN206) loading into vesicles. **f**, Cells expressing endogenous WDR7-dTAG-3xHA and stably expressing the antiporter VMAT2 were pretreated with or without dTAG13. Then, a similar assay to **a** was performed, but the cells were stained with FFN206, a fluorescent neurotransmitter analog. Fluorescence was measured by flow cytometry. **g**, The same as in **d**, but for either the *DMXL1* KO alone or for two cell clones that additionally express DMXL2-dTAG-3xHA from the endogenous locus. **h**, The same as in **f**, but for *DMXL1*-KO cells expressing endogenous DMXL2-dTAG-3xHA. **i**, The same as in **c**, but using siRNA to deplete WDR7 or ROGDI (for protein levels, see Extended Data Fig. 2f,g).

The V-ATPase holoenzyme consists of two subcomplexes: the cytosolically oriented V_1_ ATPase and the membrane-embedded V_O_ proton pump (Fig. 1a)^8^. V-ATPases operate through a rotary mechanism^9,10^. The V_1_ subcomplex contains a hexamer of alternating A and B subunits, where ATP hydrolysis at their interfaces drives the rotation of a central rotor composed of V_1_ subunits D and F and the V_O_ subunit d. The rotation of the rotor, in turn, induces rotation of the membrane-embedded c ring of the V_O_ complex, causing proton translocation at the interface between the c ring and subunit a. Peripheral stalks formed by subunits E and G anchor subunit a, preventing it from rotating along with the c ring. The V_O_ complex includes alternative isoforms of subunit a (a1–a4), each targeting the pump to a different cellular location. Specifically, a1 targets V_O_ to lysosomes^11^ as well as to presynaptic membranes and synaptic vesicles at nerve terminals^12^, a2 to the Golgi and early endosomes^13^, a3 to late endosomes and lysosomes^14^ and a4 to the plasma membrane in specialized cell types such as renal intercalated cells^12^.

V-ATPase activity can be regulated by the reversible dissociation and association of its V_1_ and V_O_ subcomplexes^15^. In *Saccharomyces cerevisiae*, V-ATPases dissociate in response to glucose deprivation^16^. When dissociated, the ATPase activity of the V_1_ subcomplex and the proton-pumping function of the V_O_ subcomplex are both auto-inhibited. On glucose readdition, V_1_ and V_O_ reassemble, a process that is facilitated by the RAVE complex (regulator of H^+^-ATPase of the vacuolar and endosomal membrane), which consists of Skp1p, Rav1p and Rav2p (refs. ^17,18^). How V-ATPase assembly is regulated in higher eukaryotes is not well understood, although some insight has been provided^19–22^.

Two subunits of the *S. cerevisiae* RAVE complex, Skp1p and Rav2p, have homologs in higher eukaryotes. SKP1 is a component of SCF complexes^23^, which regulate the ubiquitination of proteins, but its role in V-ATPase assembly is uncertain. ROGDI is homologous to Rav2p, as we and others have noticed^24–26^, but it is unclear whether it has the same function. Potential candidates for constituents of a metazoan RAVE complex include the paralogs DMXL1 and DMXL2, as well as WDR7, which are exceptionally large proteins that interact with one another^27,28^. *Drosophila* mutants of DMXL1 and DMXL2 (DMXL1/2) and WDR7 display defects similar to those observed with V-ATPase mutants, including impaired Notch signaling that results from disrupted endosomal pH^29^. DMXL1/2 and WDR7 were initially named Rabconnectin-3α and -3β, as they were identified as regulators of the small GTPase Rab3, which plays a role in vesicular trafficking^28^. However, the relevance of their interaction with Rab3 remains uncertain.

The mechanisms driving V_1_–V_O_ dissociation and reassociation in mammalian cells remain unclear (Fig. 1a). Recent studies suggest that ion gradient collapse across the lysosomal membrane triggers V_1_–V_O_ assembly^30^. The assembled V-ATPase recruits the autophagy protein ATG16L1 (refs. ^31–33^), which then promotes the conjugation of LC3/ATG8 onto phosphatidylethanolamine and phosphatidylserine in lipid bilayers^34–36^. In this noncanonical autophagy pathway, known as CASM (conjugation of ATG8 onto single membranes) or VAIL (V-ATPase-ATG16L1-induced LC3 lipidation), LC3/ATG8 is attached to lysosomal or Golgi membranes, bypassing several upstream components of canonical autophagy^37^. A key step in CASM is V_1_–V_O_ assembly^31,38^, although whether this occurs spontaneously or is catalyzed remains unknown.

In this paper, we demonstrate that DMXL1 or DMXL2, WDR7 and ROGDI form a heterotrimeric metazoan RAVE complex (mRAVE). On dissipation of the proton gradient, mRAVE catalyzes V_1_–V_O_ assembly, thereby enabling lysosomal acidification, loading of vesicles with monoaminergic neurotransmitters and recruitment of ATG16L1, which triggers CASM.

## Results

### WDR7, DMXL1/2 and ROGDI are critical for organelle acidification

We conducted an unbiased screen by adopting a previously described assay^39^ to identify proteins essential for the reacidification of lysosomes that had previously lost their proton gradient. The proton gradient was dissipated by treating cells with the selective and reversible V-ATPase inhibitor bafilomycin A1 (BafA1), which disrupts the interaction between the c ring and subunit a of the V_O_ subcomplex^40^. BafA1 is also believed to perturb the association of V_1_ with V_O_ (ref. ^30,31^). Human embryonic kidney (HEK)-293T cells were infected with lentivirus to stably express a genome-wide CRISPR–Cas9 library and then treated with BafA1. After a washout period to allow recovery, the cells were incubated with LysoTracker, a dye that fluoresces at low pH (Fig. 1b). Cells exhibiting the lowest fluorescence likely lack components required for lysosomal reacidification. Candidate components were identified by cell sorting, followed by sequencing of the CRISPR single-guide RNAs (sgRNAs) enriched in cells with low fluorescence. As expected, several subunits of the V-ATPase were identified (Fig. 1c, purple dots and Supplementary Table 1). However, the most prominent effect on reacidification was observed on disruption of *WDR7* (orange dot).

**Extended Data Fig. 1:**
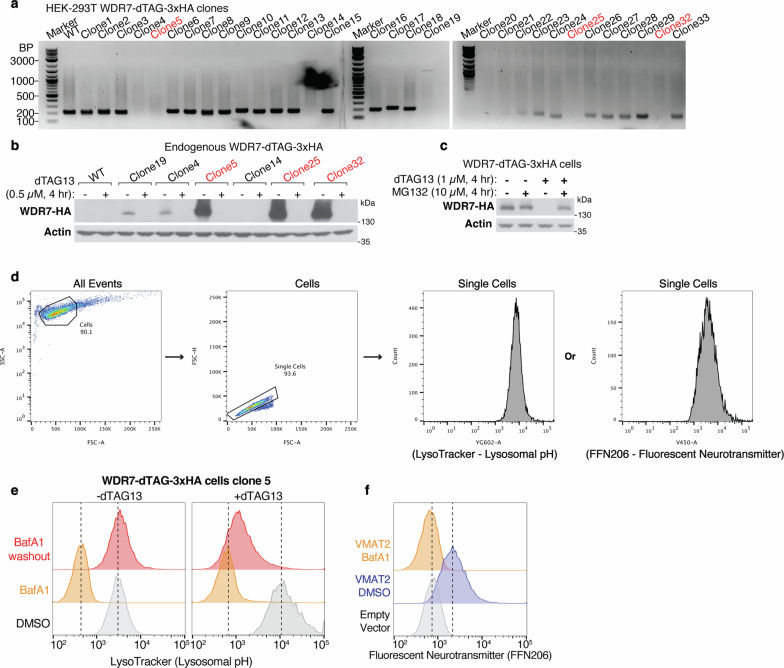
Generation of WDR7-dTAG-3xHA-expressing HEK-293T cells. (**a**) Genomic DNA from isolated clones was used as a template for polymerase chain reaction (PCR) with primers flanking the C-terminal insertion region. The absence of a band on the agarose gel indicates a homozygous insertion. Clones in red were used for the experiment in Fig. 1d. (**b**) Cells were pre-treated with or without dTAG13. Cell lysates were subjected to SDS-PAGE and immunoblotting with the indicated antibodies. Clones in red were used for the experiment in Fig. 1d. (**c**) Cells expressing WDR7-dTAG-3xHA (clone 5) were treated with dTAG13, with or without the proteasome inhibitor MG132, as indicated. Cell lysates were subjected to SDS-PAGE and immunoblotting with the indicated antibodies. (**d**) Representative schematic of the flow cytometry gating analysis used in Figs. 1c, d, f, g, h, i; 4f; Extended Data Figs. 1e, f; 2e; 7c, f. The first gate selected for cells (in the example shown 90.1% of all events), and the second gate selected for single cells (in this case 93.6%). Examples of the fluorescence intensity histograms of the single cells is shown for Lysotracker or FFN206. (**e**) Cells expressing WDR7-dTAG-3xHA from the endogenous locus (clone 5) were pre-treated with or without the degrader dTAG13. They were then treated with BafA1, the drug was removed, and the cells were recovered in fresh medium. Cells were stained with LysoTracker and analyzed by flow cytometry. (**f**) HEK-293T cells were infected with lentivirus to stably express an empty vector or VMAT2. The cells were then pre-treated with or without BafA1 and then stained with FFN206. Fluorescence was measured by flow cytometry. Source data

WDR7 is essential for cell viability^41^, so we generated a cell line enabling the acute degradation of WDR7. Sequences coding for a degradation signal (dTAG)^42^ and a 3xHA epitope tag were inserted at the 3′ end of both endogenous copies of *WDR7*, resulting in the expression of WDR7-dTAG-3xHA (Extended Data Fig. 1a,b). Cells treated with the dTAG13 compound^42^ recruit a ubiquitin ligase that triggers the rapid proteasomal degradation of the fusion protein. Several cell clones were isolated that contained two copies of the gene coding for WDR7-dTAG-3xHA; as expected, the fusion protein was degraded on dTAG13 addition in a proteasome-dependent manner (Extended Data Fig. 1c). The identified clones remained viable during the short period of dTAG13 treatment. After BafA1 washout, the clones all showed a complete defect in the reacidification of lysosomes (Fig. 1d and Extended Data Fig. 1d,e), confirming that WDR7 is necessary for restoring lysosomal acidity. These results are consistent with a previous RNAi interference (RNAi) experiment^39^.

In neurons, antiporters harness the proton electrochemical gradient generated by V-ATPases to fill synaptic vesicles with certain neurotransmitters. We reconstituted monoaminergic neurotransmitter loading in HEK-293T cells expressing WDR7-dTAG-3xHA by exogenously expressing VMAT2 (SLC18A2). This transporter exchanges protons from the lumen of synaptic vesicles for cytosolic monoamines, such as dopamine, serotonin, adrenaline or histamine (Fig. 1e)^43^. The accumulation of these neurotransmitters in vesicles can be followed by the cellular uptake of the fluorescent neurotransmitter analog FFN206 (refs. ^37,44–46^). Indeed, FFN206 accumulated in HEK-293T cells in a VMAT2-dependent manner (Extended Data Fig. 1f). The addition of BafA1 inhibited FFN206 accumulation (Extended Data Fig. 1f), confirming that a proton gradient is required for neurotransmitter loading into vesicles. After washout of BafA1, the proton gradient was restored, leading to FFN206 accumulation (Fig. 1f). In contrast, when cells were treated with dTAG13 to acutely deplete WDR7, FFN206 accumulation was blocked (Fig. 1f).

**Extended Data Fig. 2:**
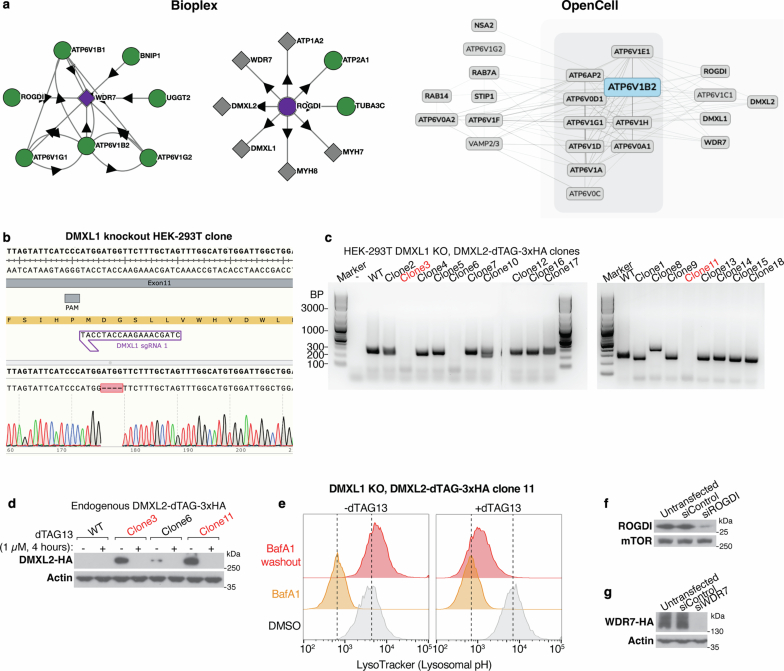
Interactions from large-scale proteomics and generation of DMXL2-dTAG-3xHA-expressing HEK-293T cells. (**a**) Interactions among mRAVE components and with V-ATPase subunits reported in proteomic studies^47,48^. (**b**) Verification of the deletion of the DMXL1 gene. Genomic DNA from an isogenic clone was used as a template for PCR with primers flanking the DMXL1 sgRNA cut site. Sanger sequencing of the PCR product shows a biallelic four nucleotide deletion within exon 11 of DMXL1. (**c**) Genomic DNA from isolated clones was used as a template for PCR with primers flanking the C-terminal insertion region. The absence of a band on the agarose gel indicates a homozygous insertion. Clones in red were used for the experiment in Fig. 1g. (**d**) Cells were pre-treated with or without dTAG13. Cell lysates were subjected to SDS-PAGE and immunoblotting with the indicated antibodies. (**e**) Cells expressing DMXL2-dTAG-3xHA in the *DMXL1* knockout background (clone 11) were pre-treated with or without the degrader dTAG13. They were then treated with BafA1, the drug was removed, and the cells were recovered in fresh medium. Cells were stained with LysoTracker and analyzed by flow cytometry. (**f**) Cells were transfected with ROGDI-targeting siRNA or control siRNA, as indicated. ROGDI levels were determined two days after transfection by immunoblotting. mTOR levels served as a loading control. (**g**) As in (**f**), but using siRNA targeting WDR7 in WDR7-dTAG-3xHA cells. Actin served as a loading control. Source data

Large-scale proteomics studies have reported that WDR7, DMXL1/2 and ROGDI coimmunoprecipitate with each other and with V-ATPase subunits^39,47,48^ (Extended Data Fig. 2a), suggesting that DMXL1/2 and ROGDI may also have a role in regulating the V-ATPase. DMXL1 and DMXL2 are close homologs and are expected to have identical functions. Both proteins are expressed in almost all cells^49^ and are present at about equal levels in HEK-293T cells^47^. We again used acute degradation to test the role of DMXL1 and DMXL2 in the reacidification of lysosomes. We first generated CRISPR-knockout (KO) cells lacking the *DMXL1* gene (Extended Data Fig. 2b). Then, sequences coding for a dTAG and a 3xHA epitope tag were inserted at the 3′ end of both endogenous copies of *DMXL2*, resulting in the expression of DMXL2-dTAG-3xHA (Extended Data Fig. 2c,d). In cells lacking *DMXL1*, lysosomes were readily reacidified following BafA1 washout (Fig. 1g), consistent with the expectation that loss of *DMXL1* or *DMXL2* alone should have no effect, and with the observation that neither gene scored in the genome-wide screen (Fig. 1c). Indeed, when DMXL2 was additionally depleted by its acute degradation after addition of dTAG13, lysosomal reacidification and FFN206 loading into VMAT2-containing vesicles were abolished (Fig. 1g,h and Extended Data Fig. 2e). The reported effect of DMXL1 depletion alone on lysosomal pH may perhaps be explained if this isoform is expressed at higher levels than DMXL2 in the chosen cell lines^39,50^.

We also tested the role of ROGDI in lysosomal reacidification after BafA1 washout. RNAi-mediated depletion of ROGDI had a modest effect on lysosomal pH (Fig. 1i and Extended Data Fig. 2f,g), consistent with it being nonessential for cell viability^41^. These experiments show that WDR7 and DMXL1/2 are necessary for lysosomal acidification and the packaging of vesicles with monoaminergic neurotransmitters, whereas ROGDI plays a facilitating role.

### WDR7, DMXL2 and ROGDI form a metazoan RAVE complex

**Fig. 2:**
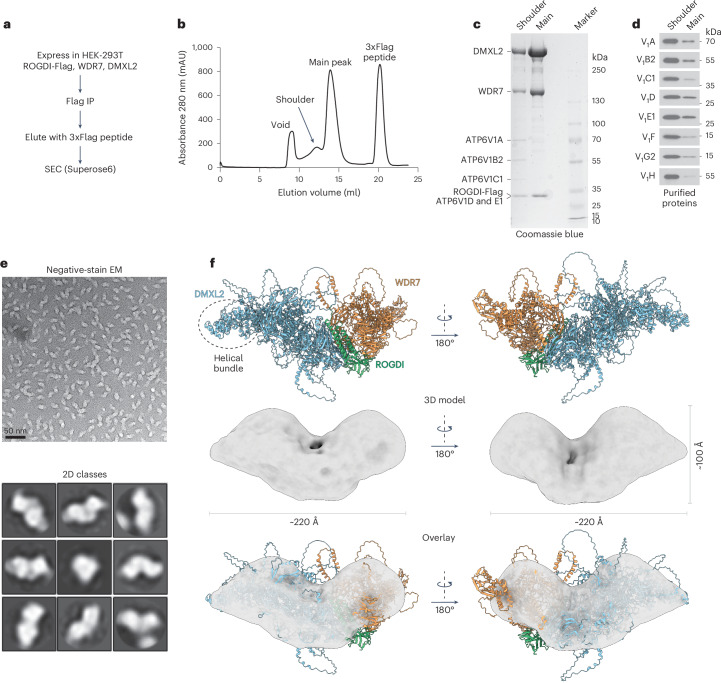
WDR7, DMXL1 or DMXL2 and ROGDI form a stoichiometric mRAVE complex. **a**, Purification protocol for the mRAVE complex. **b**, SEC profile (absorbance at 280 nm). Fractions containing the main or shoulder peaks were pooled. **c**, Aliquots of the main and shoulder peaks were subjected to SDS–polyacrylamide gel electrophoresis (SDS–PAGE) and Coomassie blue staining. Molecular weight markers were run and their size in kDa is shown on the right. **d**, Immunoblotting of the two peaks with antibodies to the indicated subunits of the V_1_ complex. **e**, Negative-stain electron microscopy (EM) of the purified mRAVE complex. Top: individual particles. Bottom: 2D averages. **f**, Top: AlphaFold3-predicted model of the mRAVE complex in two different views. Middle and bottom: 3D model derived from the negative-stain data in **e** overlaid with the AlphaFold model. Source data

**Extended Data Fig. 3:**
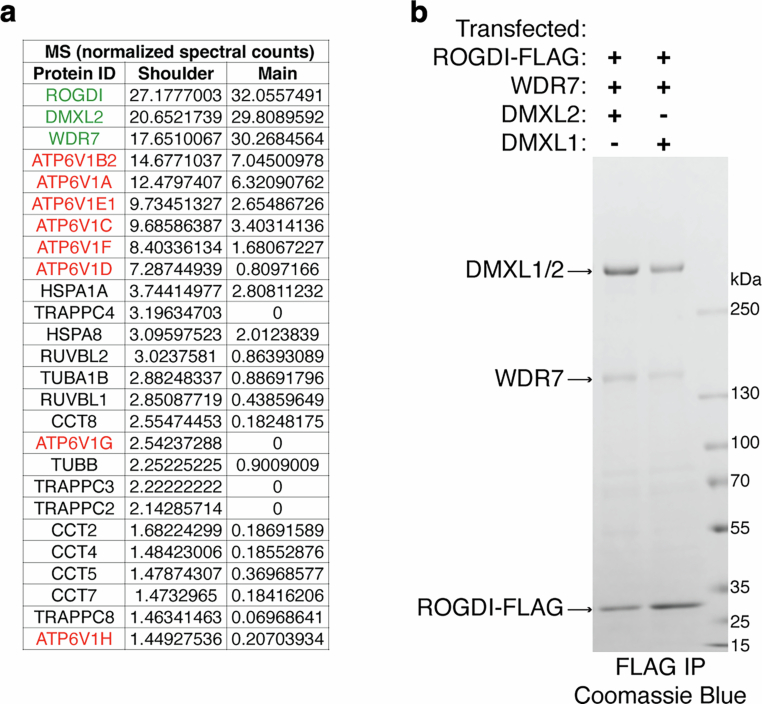
Analysis of purified mRAVE fractions by mass spectrometry and demonstration that DMXL1 and DMXL2 form similar complexes. (**a**) Proteins in the main and shoulder peaks in Fig. 2b were digested with trypsin, and the peptides were analyzed by mass spectrometry. Shown are the spectral counts for the most enriched proteins, normalized to the molecular weight. (**b**) Plasmids encoding DMXL1 or DMXL2, WDR7, and ROGDI-FLAG were transfected in a 13:6:1 molar ratio. Cell lysates were subjected to immunoprecipitation with FLAG antibodies, and the samples were analyzed by SDS-PAGE and Coomassie blue staining. Source data

To investigate whether the three proteins form a stable, stoichiometric complex, we cotransfected HEK-293T cells with plasmids encoding human DMXL2, WDR7 and ROGDI-Flag (Fig. 2a). A detergent extract was subjected to immunoprecipitation (IP) with Flag antibodies, followed by size-exclusion chromatography (SEC). Two major peaks were observed (Fig. 2b). The main peak contained DMXL2, WDR7 and ROGDI-Flag in approximately stoichiometric quantities (Fig. 2c). A smaller shoulder peak contained these three proteins and subunits of the V_1_ complex. Immunoblotting and mass spectrometry analysis confirmed that the shoulder peak was enriched in all eight V_1_ subunits relative to the main peak (Fig. 2d) and notably lacked V_O_ subunits (Extended Data Fig. 3a and Supplementary Table 2). Similar results were obtained when DMXL2 was replaced with DMXL1 (Extended Data Fig. 3b). These findings suggest that DMXL1 or DMXL2, WDR7 and ROGDI form a stable, stoichiometric complex, which we refer to as the mRAVE. Our results also indicate that, at a steady state, mRAVE interacts with the V_1_ but not the V_O_ complex.

**Extended Data Fig. 4:**
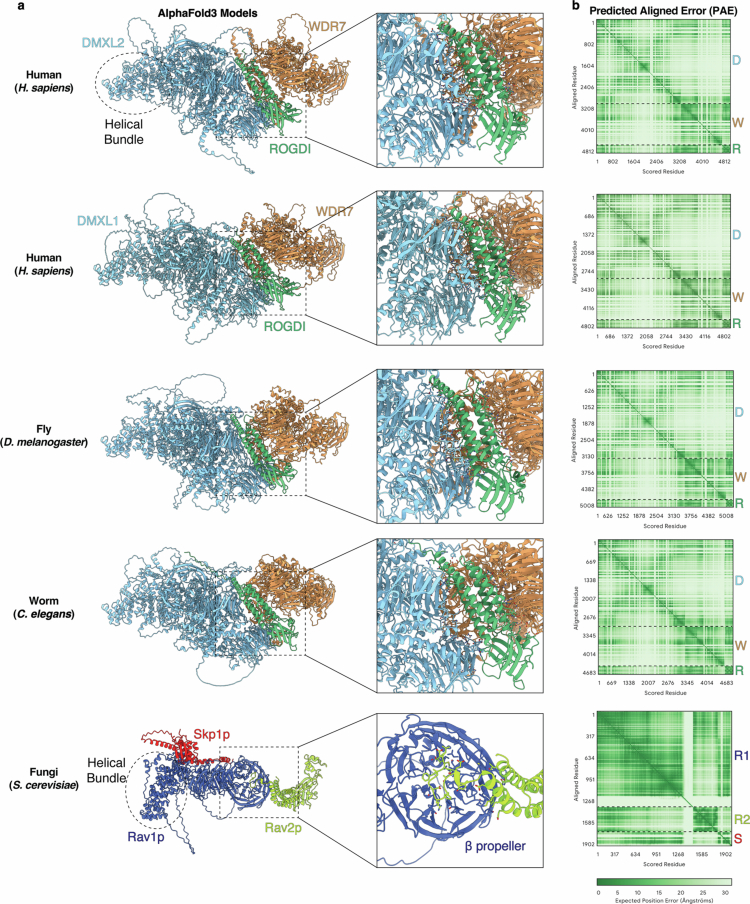
AlphaFold3-predicted models of RAVE complexes from different species. (**a**) AlphaFold3 models of the indicated RAVE complexes. The boxed regions are shown in a magnified view on the right. The helical bundle of DMXL2/Rav1p is highlighted. (**b**) Predicted Aligned Error (PAE) plots for each model, with a darker green color showing increased prediction confidence. D, DMXL; W, WDR7; R, ROGDI; R1, Rav1p; R2, Rav2p; S, Skp1p.

The size of mRAVE particles in negative-stain electron microscopy images is consistent with the presence of one copy of each of its three subunits (Fig. 2e, top), in agreement with mass spectrometry analysis that showed approximately equal amounts of tryptic peptides when corrected for the size of the three components (Extended Data Fig. 3a and Supplementary Table 2). In two-dimensional (2D) class averages, the particles resemble ‘angel wings’ (Fig. 2e, bottom). AlphaFold3 (ref. ^51^) predicts that DMXL2 and WDR7 form the wings on either side of the elongated structure (Fig. 2f). ROGDI is predicted to be located between these two proteins, acting as a linker. The same general structure is predicted for the orthologous complexes in flies and worms, suggesting that mRAVE is conserved among metazoans (Extended Data Fig. 4a,b).

**Fig. 3:**
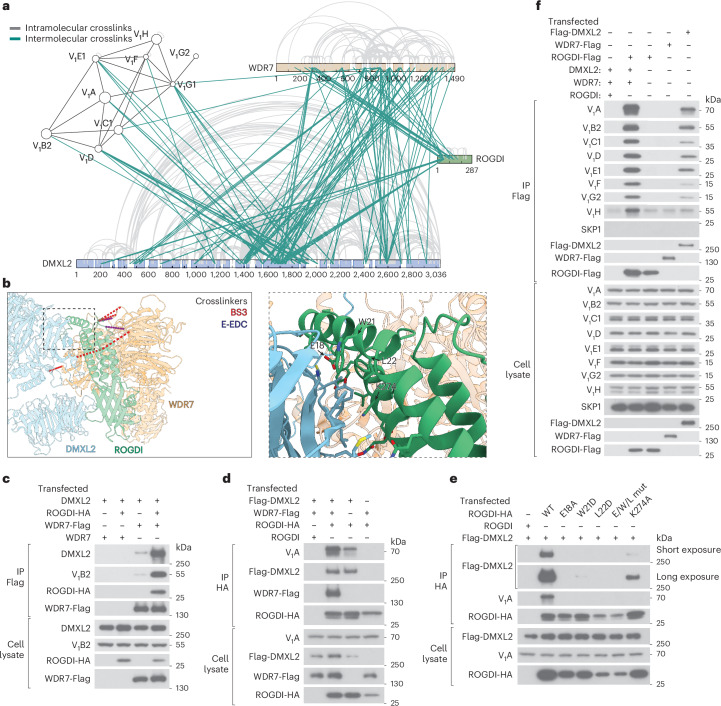
Interactions among mRAVE components and with the V_1_ complex. **a**, The main and shoulder fractions from Fig. 2b were individually treated with two bifunctional crosslinkers (BS3 and E-EDC), and crosslinked tryptic peptides were identified by mass spectrometry. Each line is an observed crosslink between the indicated regions of the components. Intra- and intermolecular crosslinks are shown in gray and green, respectively. **b**, AlphaFold3-predicted mRAVE model, with the highest confidence BS3 and E-EDC crosslinks between the components indicated by red and purple broken lines, respectively. The square indicated by broken lines is magnified on the right with ROGDI residues at the interface labeled. **c**, HEK-293T cells were transfected with cDNAs coding for WDR7-FLAG, ROGDI-HA and untagged DMXL2 or WDR7, as indicated. Cell lysates were subjected to IP with Flag antibodies. The samples were analyzed by SDS–PAGE and immunoblotting with the indicated antibodies. **d**, The same as in **c**, with expression of the indicated proteins. ROGDI-HA was immunoprecipitated with HA antibodies, and coprecipitated proteins were analyzed by SDS–PAGE and immunoblotting with the indicated antibodies. **e**, The same as in **d**, using ROGDI mutants in which residues at the interface to DMXL2 (**b**) were altered. E/W/L mut is a combination of the three single mutants. **f**, The same as in **c**, with expression of the indicated proteins. Flag-immunoprecipitates were analyzed by SDS–PAGE and immunoblotting with the indicated antibodies. Source data

To validate the AlphaFold3-predicted structure, we performed crosslinking with two different bifunctional reagents bis(sulfosuccinimidyl)suberate (BS3) and 1-ethyl-3-(3-dimethylaminopropyl)carbodiimide (E-EDC), followed by mass spectrometry analyses of crosslinked tryptic peptides (Fig. 3a and Supplementary Table 3). The results are consistent with ROGDI being situated between DMXL2 and WDR7 (Fig. 3b). Transfection of Flag-tagged WDR7 together with untagged DMXL2 in the absence of ROGDI showed that small amounts of DMXL2 were precipitated with Flag antibodies (Fig. 3c), indicating that WDR7 and DMXL2 interact weakly with one another. However, consistent with the structural model, the coexpression of the linker protein ROGDI strongly enhanced the binding of DMXL2 to WDR7-FLAG (Fig. 3c). ROGDI interacted strongly with DMXL2 even in the absence of WDR7 (Fig. 3d). In contrast, ROGDI associated with WDR7 only in the presence of DMXL2 (Fig. 3d). Most ROGDI mutations at the predicted interface with DMXL2 abolished the binding, whereas one K274A had a partial effect (Fig. 3e). These results further validate the AlphaFold3 model.

The crosslinking mass spectrometry experiments also revealed that DMXL2 is responsible for almost all the crosslinks between mRAVE and the V_1_ complex (Fig. 3a). To validate this finding, we expressed Flag-tagged versions of the mRAVE subunits either individually or together. IP with Flag antibodies showed that Flag-DMXL2 is sufficient to precipitate all eight V_1_ subunits (Fig. 3f). However, the interaction with V_1_ was much stronger when DMXL2 was in complex with WDR7 and ROGDI. These results confirm that DMXL2 mediates the interaction with the V_1_ complex and indicate that the other two subunits stabilize DMXL2 in the intact mRAVE complex. It should be noted that the SKP1 protein was not precipitated (Fig. 3f). Thus, mRAVE does not contain SKP1, unlike the yeast RAVE complex.

**Extended Data Fig. 5:**
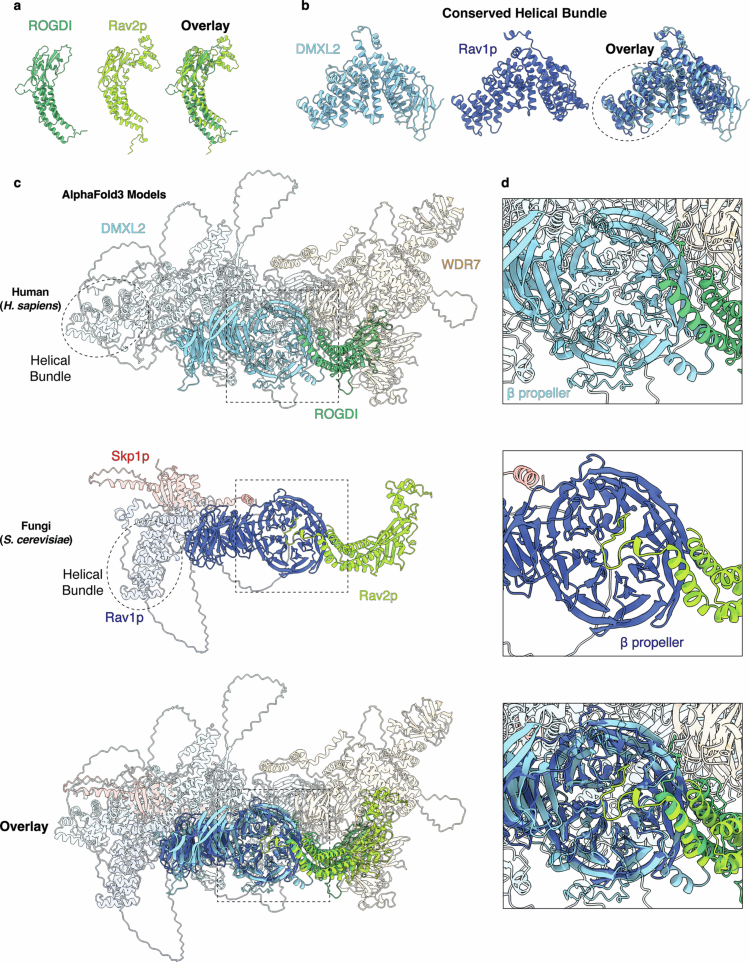
Comparison of the metazoan and yeast RAVE complexes. (**a**) AlphaFold predictions of ROGDI and Rav2p are shown individually and overlayed. (**b**) The terminal helical bundle domain of DMXL2 and Rav1p are shown individually and overlayed. (**c**) AlphaFold3 models of the indicated RAVE complexes, highlighting the β propellers and ROGDI or Rav2p. The square indicated by broken lines shows the interactions. (**d**) Magnification of the squares in (**c**).

Some features of mRAVE are similar to those of the yeast complex. Mammalian ROGDI and yeast Rav2p are predicted to have roughly superimposable structures (Extended Data Fig. 5a). DMXL1/2 and yeast Rav1p both contain terminal helical bundle domains that can be well aligned (Extended Data Fig. 5b). Moreover, both proteins have two superimposable β-propeller domains, and one of these domains interacts similarly with the respective partners ROGDI and Rav2p (Extended Data Fig. 5c,d).

Nevertheless, the overall structures of mRAVE and yeast RAVE are very different. Whereas mRAVE resembles ‘angel wings’, the yeast RAVE complex has an L-shape (Extended Data Fig. 4), as reported in a recent cryo-EM structure of the yeast complex^25^. ROGDI serves as a linker between DMXL1/2 and WDR7, whereas the related Rav2p is located at the end of the L-shaped yeast complex. SKP1 is found only in the yeast complex. WDR7 is an essential component of the metazoan complex but is absent in yeast, suggesting that it was added more recently during evolution. Indeed, the two mRAVE subunits (ROGDI and DMXL1/2) with similarity to the yeast components interact more strongly with one another than with WDR7 (Fig. 3d). Given that V-ATPases are highly conserved from yeast to humans, it is surprising that mRAVE has diverged considerably in structure and composition from its ancestral counterpart.

### DMXL2 binds directly to subunits A and D of the V_1_ complex

**Fig. 4:**
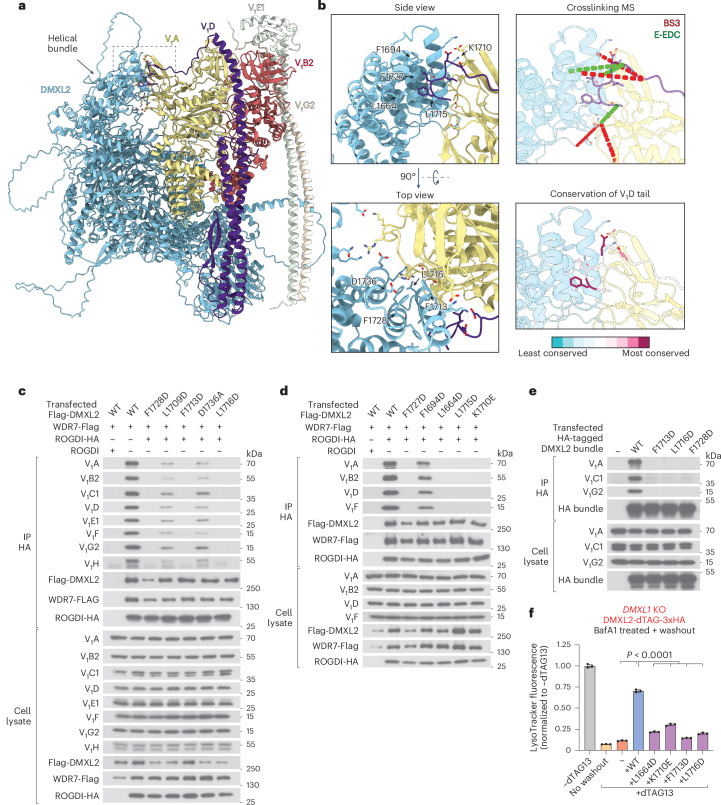
DMXL2 interacts with subunits A and D of the V_1_ complex. **a**, AlphaFold3-predicted interactions of DMXL2 with subunits of the V_1_ complex, using a model containing single copies of subunits A, B2, D, E1 and G2. The numbers following the letters refer to the dominant isoforms in HEK-293T cells. The square indicated by broken lines contains the interactions between the helical bundle of DMXL2 with subunits A and D of the V_1_ complex. **b**, Top left: magnification of the square in **a** (subunits A and D in yellow and purple, respectively). The indicated amino acids of DMXL2 were mutated. Bottom left: the same as in the top panel, but in a rotated view. Top right: observed crosslinks with BS3 or E-EDC shown as red and green broken lines, respectively. Bottom right: conservation of the C-terminal tail of subunit D calculated by ConSurf^68^ (scale at the bottom). **c**, Flag-tagged WT DMXL2 or the indicated mutants carrying substitutions at the interface with subunit A of the V_1_ complex were coexpressed with WDR7-Flag and untagged or HA-tagged ROGDI. Cell lysates were subjected to IP with HA antibodies. The samples were analyzed by SDS–PAGE and immunoblotting with the indicated antibodies. **d**, The same as in **c**, but with DMXL2 mutants carrying substitutions at the interface with subunit D of the V_1_ complex. **e**, The same as in **c**, but with cells expressing only the WT or mutant helical bundle of DMXL2. **f**, *DMXL1*-KO cells expressing endogenous DMXL2-dTAG-3xHA were engineered to stably overexpress WT or mutant HA-DMXL2 defective in the interaction with the V_1_ complex (for expression levels, see Extended Data Fig. 6d). The cells were treated with or without the degrader dTAG13. After treatment with BafA1, the drug was removed, and the cells were recovered in fresh medium. Cells were then stained with LysoTracker and analyzed by flow cytometry. Shown are the means and standard deviation of the median fluorescence intensity from *n* = 3 biological replicates. Data were analyzed using ordinary one-way analysis of variance followed by Dunnett’s multiple-comparisons test (*P* < 0.0001). MS, mass spectrometry. Source data

We used AlphaFold3 to predict the interactions of DMXL2 with the V_1_ complex. The model included DMXL2 and single copies of the ATP-hydrolyzing subunits A and B2, the central rotor subunit D and the peripheral stalk subunits E1 and G2 (the numbers following the letters indicate the dominant isoforms in HEK-293T cells). According to the predicted model, DMXL2 uses its terminal helical bundle domain to interact with subunit A and the C-terminal tail of subunit D of the V_1_ complex (Figs. 2f and 4a). Both interaction surfaces contain primarily hydrophobic amino acids and are consistent with numerous intermolecular crosslinks observed in our mass spectrometry experiments (Fig. 4b and Supplementary Table 3).

**Extended Data Fig. 6:**
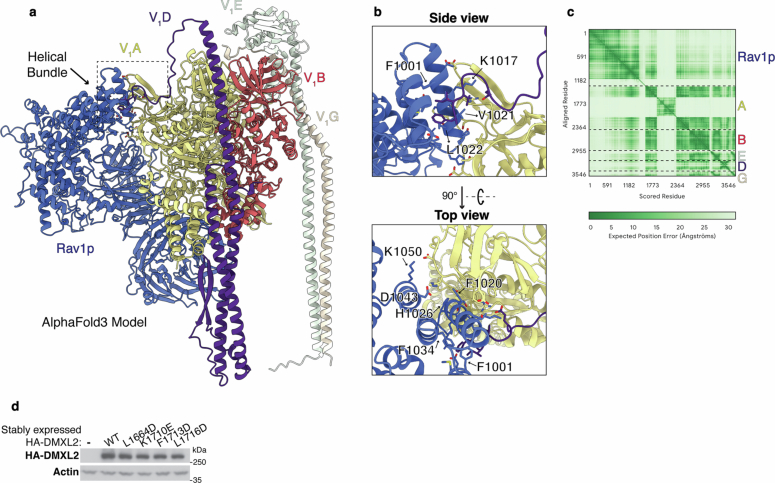
AlphaFold3-predicted model of Rav1p bound to subunits A and D of the V_1_ complex. (**a**) AlphaFold3-predicted interactions of Rav1p with subunits of the yeast V_1_ complex, using a model containing single copies of subunits A, B, D, E, and G. The square indicated by broken lines shows the interactions between the helical bundle of Rav1p with subunits A and D of the V_1_ complex. (**b**) Upper panel: magnification of the square in (**a**) (subunits A and D in yellow and purple, respectively). Lower panel: as in the upper panel, but in a rotated view. (**c**) Predicted Aligned Error (PAE) plot, with a darker green color showing increased prediction confidence. (**d**) *DMXL1* knockout cells expressing endogenous DMXL2-dTAG-3xHA were engineered to stably overexpress wild-type or mutant HA-DMXL2 defective in the interaction with the V_1_ complex. Cell lysates were analyzed by SDS-PAGE followed by immunoblotting with HA antibodies. Blotting for actin served as a loading control. Source data

The C-terminal tail of subunit D is invisible in all reported V-ATPase structures, likely because it is flexible. However, the sequence of the tail is highly conserved (Fig. 4b), suggesting that both mRAVE and the yeast complex interact with subunit D. Indeed, AlphaFold3 also predicts this interaction for the helical bundle domain of yeast Rav1p (Extended Data Fig. 6a–c), even though it was not reported in a recent paper describing the structure of the yeast RAVE–V_1_ complex^25^.

To further validate the predicted interactions between DMXL2 and the V_1_ subunits, we first introduced mutations at the DMXL2 interface with subunit A. Flag-tagged DMXL2 mutants were expressed alongside wild-type (WT) WDR7-Flag and ROGDI-HA, and cell extracts were subjected to IP with hemagglutinin (HA) antibodies (Fig. 4c). All mutations either diminished or abolished the interaction with the V_1_ complex while leaving the mRAVE complex intact. Similar experiments were performed with DMXL2 mutants predicted to disrupt the interaction with subunit D of V_1_ (Fig. 4d). Except for the F1694D mutation, all substitutions abolished the binding of mRAVE to the V_1_ complex.

To further confirm that the helical bundle of DMXL2 mediates the interaction with V_1_, we expressed the domain alone as an HA-tagged protein and performed IP with HA antibodies (Fig. 4e). The WT domain coprecipitated V_1_ subunits, whereas mutants disrupting the interaction with subunit A failed to do so. Together, these results demonstrate that the terminal bundle domain of DMXL2 directly interacts with subunits A and D of the V_1_ complex. Both interactions are required for the binding of mRAVE to V_1_.

To test whether the interaction of DMXL2 with subunits A and D of the V_1_ complex is functionally important, we overexpressed WT or mutant DMXL2 in cells lacking *DMXL1*and expressing DMXL2-dTAG-3xHA from the endogenous locus. The transgenes were introduced into the genome using a transposon-based system, as the large size of DMXL2 prevented its efficient packaging into lentivirus particles. WT DMXL2 almost completely rescued the reacidification of lysosomes after degradation of DMXL2-dTAG-3xHA and BafA1 washout (Fig. 4f and Extended Data Fig. 6d). In contrast, reacidification was impaired in cells expressing the DMXL2 mutants. These results show that the interaction of DMXL2 with both subunits A and D of the V_1_ complex is functionally important.

### mRAVE catalyzes V_1_–V_O_ assembly on dissipation of proton gradients

**Extended Data Fig. 7:**
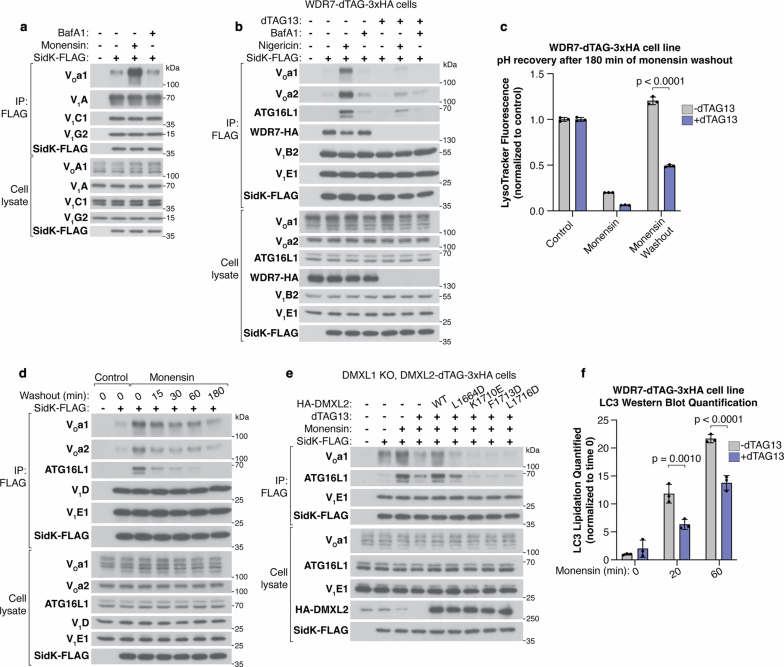
Testing V_1_-V_O_ assembly. (**a**) HEK-293T cells were treated with or without monensin or BafA1, as indicated. Purified SidK-FLAG was added to cell lysates to capture the V_1_ complex, and the lysates were subjected to immunoprecipitation with FLAG antibodies. Samples were analyzed by SDS-PAGE and immunoblotting with the indicated antibodies. (**b**) Cells expressing WDR7-dTAG-3xHA from the endogenous locus were treated with the degrader dTAG13 and the ionophore nigericin, as indicated. The assembly of V_1_ with Vo was followed as in (**a**). (**c**) Cells expressing WDR7-dTAG-3xHA were treated with or without the degrader dTAG13. After treatment with monensin, the drug was removed, and the cells were recovered in fresh medium. Cells were then stained with LysoTracker and analyzed by cell sorting. Shown are the means and standard deviation of the median fluorescence intensity (MFI) from n = 3 biological replicates. Data were analyzed using ordinary one-way ANOVA followed by Tukey’s multiple-comparisons test (p < 0.0001). (**d**) Cells were treated with monensin, the drug was washed out, and the disassembly of the V-ATPase into V_1_ and V_O_ was followed over time using the assay described in (**a**). **(e)** *DMXL1* knockout cells expressing DMXL2-dTAG-3xHA from the endogenous locus were engineered to stably overexpress wild-type or mutant HA-DMXL2 defective in the interaction with the V_1_ complex. The cells were treated with or without dTAG13 and monensin, as indicated. The assembly of V_1_ with Vo was followed as in (**a**). (**f**) Cells expressing WDR7-dTAG-3xHA were pre-treated with or without dTAG13. Then, the cells were treated with or without monensin for the indicated periods. Cell lysates were analyzed by SDS-PAGE and immunoblotting with the indicated antibodies. LC3 lipidation was quantified, and actin was used as a loading control. Shown are means and standard deviation from n = 3 biological replicates. Data were analyzed using ordinary one-way ANOVA followed by Tukey’s multiple-comparisons test (p = 0.0010 or p < 0.0001). Source data

Next, we studied the role of mRAVE in the assembly of V_1_ with V_O_. Previous studies have suggested that drugs that dissipate the proton gradient of lysosomes lead to the assembly of V_1_ with V_O_ (ref. ^30^). Therefore, we treated cells with BafA1, a drug that abolishes the proton gradient by directly inhibiting the V-ATPase, and with monensin, a proton–sodium ionophore that disrupts the proton gradient but does not inhibit the V-ATPase. The assembly of V_1_ with V_O_ was measured by pulling on the V_1_ complex with Flag-tagged SidK^52,53^, an effector protein of *Legionella pneumophila* that binds tightly to V_1_. Purified SidK-Flag was added to cell lysates and the coprecipitation of V_O_ was determined by immunoblotting. The results show that monensin, but not BafA1, induced the assembly of V_1_ with V_O_ (Extended Data Fig. 7a). We therefore used monensin to study the role of mRAVE in the assembly of V_1_ with V_O_.

**Fig. 5:**
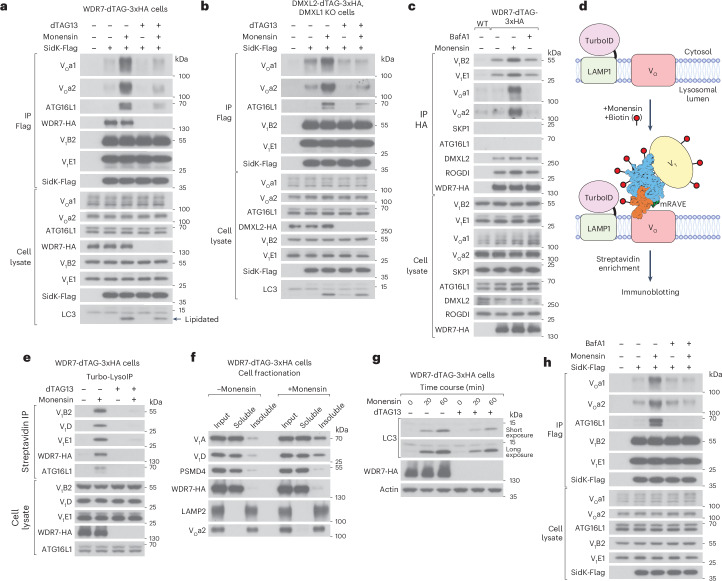
mRAVE assembles V_1_ and V_O_ on lysosomes on dissipation of the proton gradient. **a**, Cells expressing WDR7-dTAG-3xHA were pretreated with or without dTAG13. Then, the cells were treated with or without monensin, as indicated. Purified SidK-Flag was added to cell lysates to capture the V_1_ complex, and the lysates were subjected to IP with Flag antibodies. Samples were analyzed by SDS–PAGE and immunoblotting with the indicated antibodies. **b**, The same as in **a**, but with *DMXL1*-KO cells expressing DMXL2-dTAG-3xHA from the endogenous locus. **c**, WT or WDR7-dTAG-3xHA-expressing cells were treated with BafA1 or monensin. Cell lysates were subjected to IP with HA antibodies. The samples were analyzed by SDS–PAGE and immunoblotting with the indicated antibodies. **d**, Schematic showing the protocol of the Turbo-LysoIP experiment. **e**, Cells expressing WDR7-dTAG-3xHA and stably expressing a LAMP1-TurboID fusion were pretreated with dTAG13. Where indicated, the cells were treated with monensin for 30 min, followed by an additional 30 min in the presence of biotin to label lysosomal surface proteins. After cell lysis, biotinylated proteins were isolated with streptavidin beads. Bound proteins were analyzed by SDS–PAGE and immunoblotting with the indicated antibodies. **f**, Cells expressing WDR7-dTAG-3xHA were treated with or without monensin. A cell lysate generated without detergent was subjected to ultracentrifugation. The supernatant (soluble) and pellet (insoluble) fractions were analyzed by SDS–PAGE and immunoblotting with the indicated antibodies. **g**, Cells expressing WDR7-dTAG-3xHA were pretreated with or without dTAG13. Then, the cells were treated with or without monensin for the indicated time periods. Cell lysates were analyzed by SDS–PAGE and immunoblotting with the indicated antibodies. **h**, The same as in **a**, but WT HEK-293T cells were pretreated with 200 nM BafA1 for 45 min and then treated with or without 100 µM monensin for 1 h. Source data

To test whether mRAVE is required for the monensin-induced assembly of V_1_ with V_O_, we used cells expressing WDR7-dTAG-3xHA (Fig. 5a). In the absence of dTAG13 and monensin, SidK-Flag coprecipitated the V_1_ complex, but only small amounts of the V_O_ complex (Fig. 5a). Monensin-induced V_1_–V_O_ assembly, but the association was largely abolished in cells acutely depleted of WDR7 (Fig. 5a). A WDR7-dependent association of V_1_ with V_O_ was also observed when the proton gradient was dissipated by nigericin, a proton–potassium ionophore (Extended Data Fig. 7b), suggesting that mRAVE plays a general role in the assembly of the V-ATPase on proton gradient collapse. Monensin washout experiments showed that cells depleted of WDR7 also had a significant defect in the restoration of the proton gradient (Extended Data Fig. 7c). Taken together, these results indicate that V-ATPase complexes assemble after the dissipation of the proton gradient and are crucial for restoring lysosomal acidity when cells return to normal conditions. Consistent with this model, V_1_ dissociated from V_O_ during the recovery phase (Extended Data Fig. 7d).

The monensin-induced association of V_1_ with V_O_ also depends on DMXL1/2, as the association was abolished when endogenous DMXL2-dTAG-3xHA was acutely depleted in *DMXL1*-KO cells (Fig. 5b). The overexpression of WT DMXL2 rescued the assembly, whereas the overexpression of V_1_ binding-deficient mutants did not (Extended Data Fig. 7e).

To test whether mRAVE mediates the interaction between V_1_ and V_O_, detergent extracts of cells expressing WDR7-dTAG-3xHA were subjected to IP with HA antibodies, and WDR7-associated proteins were detected by immunoblotting (Fig. 5c). In untreated cells, subunits of both the mRAVE and V_1_ complexes were precipitated, but not the V_O_ complex, represented by the a1 or a2 subunits (Fig. 5c). These results confirm that, in untreated cells, mRAVE is bound to V_1_ but not V_O_. However, on treatment with monensin, HA-tagged WDR7 coprecipitated not only mRAVE and V_1_ subunits but also the V_O_ subunits a1 and a2 (Fig. 5c). Again, this effect was not observed with the V-ATPase inhibitor BafA1. Thus, monensin (but not BafA1) treatment promotes the formation of a supercomplex containing mRAVE, V_1_ and V_O_. Since the a1-containing V_O_ complex is localized to lysosomes and the a2-containing complex to the Golgi, these results indicate that the supercomplex can form on different organelles. Together, these findings demonstrate that mRAVE catalyzes V_1_–V_O_ assembly following the dissipation of proton gradients.

The mRAVE-dependent assembly of V_1_ with V_O_ can occur on the lysosomal surface. This conclusion is based on experiments in which we stably expressed a fusion of the lysosomal membrane protein LAMP1 with TurboID, a promiscuous biotin ligase^54–56^. Cells expressing WDR7-dTAG-3xHA were treated with monensin, followed by the addition of biotin to label proteins in close proximity to the lysosomal membrane (Fig. 5d). Biotinylated proteins were isolated using streptavidin beads and analyzed by immunoblotting. In the absence of dTAG13, monensin treatment considerably increased the amounts of WDR7 and V_1_ complexes on the lysosomal surface, indicating that the complex of mRAVE and V_1_ is recruited to lysosomes when the proton gradient is dissipated. However, after dTAG13-induced depletion of WDR7, the recruitment of V_1_ to the lysosomal surface was largely abolished (Fig. 5e). These findings confirm that a supercomplex forms on the lysosomal surface on dissipation of the proton gradient and that mRAVE is necessary for the assembly of V_1_ with V_O_ on the membrane. At steady state, only a small percentage of mRAVE is present on membranes, even in the presence of monensin; most mRAVE localizes to the cytosol, as shown by cell fractionation experiments (Fig. 5f). Thus, mRAVE is recruited transiently to the lysosomal surface to catalyze V-ATPase assembly.

### mRAVE enables ATG16L1 recruitment and CASM

In line with previous studies, we found that the key autophagy component ATG16L1 coprecipitated with SidK-Flag after monensin treatment, that is, on assembly of V_1_ with V_O_ (Fig. 5a,b)^38^. In cells acutely depleted of WDR7 or DMXL1/2, less ATG16L1 was coprecipitated with SidK-Flag (Fig. 5a,b). As expected, DMXL2 mutants defective in V_1_ binding and V-ATPase assembly resulted in reduced ATG16L1 coprecipitation (Extended Data Fig. 7e). Depletion of WDR7 also inhibited the recruitment of ATG16L1 to the lysosomal surface (Fig. 5e). These results show that efficient recruitment of ATG16L1 requires the mRAVE-mediated assembly of the V-ATPase.

Even after depletion of WDR7 or DMXL1/2, monensin treatment caused some ATG16L1 to be recruited to the V-ATPase. A possible explanation is that, on dissipation of the proton gradient, ATG16L1 can also bind to preexisting V-ATPase complexes. This assumption would explain why acute mRAVE-depletion attenuated, but did not abolish, ATG16L1-triggered CASM, that is the lipidation of LC3/ATG8 (Fig. 5a,b,g and Extended Data Fig. 7f). We tested this hypothesis by treating cells with BafA1 to disrupt preexisting V-ATPase complexes. Indeed, in the presence of BafA1, monensin treatment did not cause V_1_–V_O_ assembly and ATG16L1 recruitment (Fig. 5h), consistent with previous reports that BafA1 abolishes the monensin-induced LC3 lipidation in cells defective in canonical autophagy^31,57^. Thus, ATG16L1 seems to be recruited to either preexisting or newly assembled V-ATPase complexes when the proton gradient across the membrane is dissipated, and can then trigger CASM. mRAVE and ATG16L1 probably bind to the V-ATPase in a mutually exclusive manner. Whereas both components could be retrieved with the V_1_ complex via SidK (Fig. 5a) and were found on the lysosomal surface (Fig. 5e), pulling on endogenous WDR7 did not precipitate any ATG16L1 (Fig. 5c). Thus, some assembled V-ATPase molecules are associated with either mRAVE or ATG16L1, but no molecules seem to contain both.

## Discussion

We demonstrate that DMXL1 or DMXL2, WDR7 and ROGDI form a stoichiometric complex, which we name the mRAVE. mRAVE is essential for V_1_–V_O_ assembly and plays a key role in lysosomal acidification, vesicular packaging of monoaminergic neurotransmitters and CASM.

**Fig. 6:**
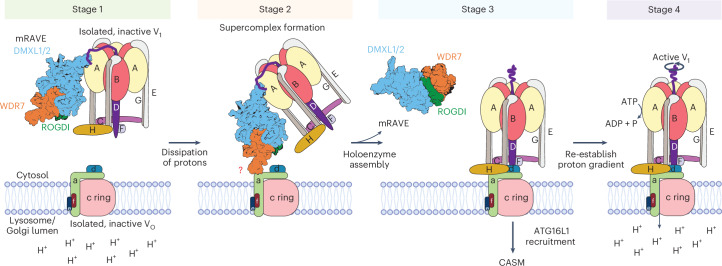
Model of V-ATPase assembly by mRAVE. Stage 1: mRAVE interacts in the cytosol with subunits A and D of the isolated, inactive V_1_ complex using the terminal helical bundle domain of DMXL1/2. Stage 2: on dissipation of the proton gradient by ionophores, influenza M2 proton channel, STING agonists or TRPML1 agonists, a supercomplex containing mRAVE, V_1_ and V_O_ forms on the lysosomal or Golgi membrane. We hypothesize that the V_O_ is the proton sensor and changes its conformation to recruit mRAVE. Stage 3: on dissociation of mRAVE, V_1_ and V_O_ assemble into the V-ATPase holoenzyme. If the proton gradient remains dissipated, ATG16L1 is recruited to the assembled V-ATPase and triggers CASM. Stage 4: the proton gradient is re-established. V_O_ would return to its original conformation, causing some V-ATPase molecules to dissociate into V_1_ and V_O_ subcomplexes, thus establishing the steady-state situation. The proton gradient may fluctuate in untreated cells, causing the reversible mRAVE-dependent assembly of the V-ATPase.

**Extended Data Fig. 8:**
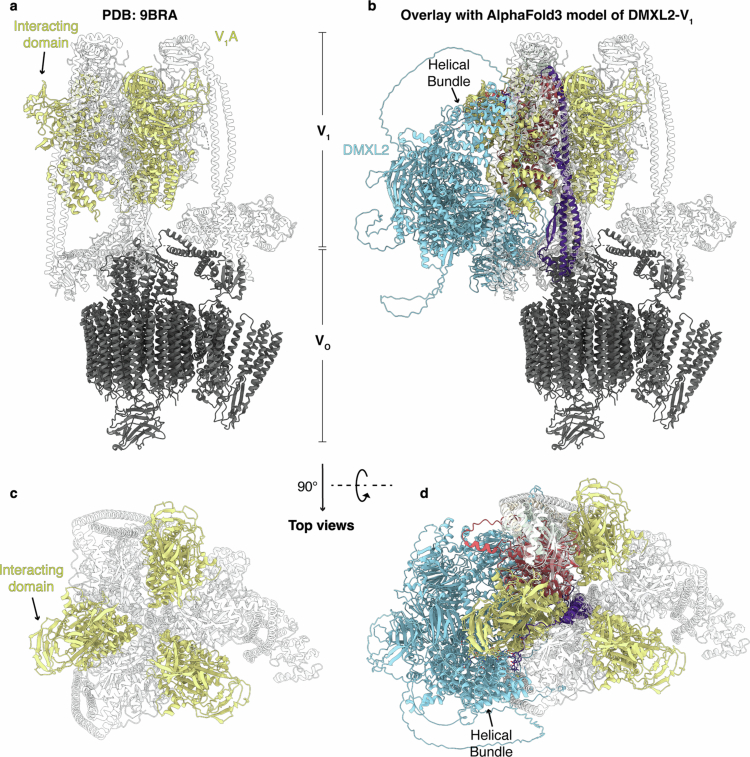
Predicted structure of mRAVE bound to the V-ATPase holoenzyme. **(a)** Cryo-EM structure of the V-ATPase holoenzyme (PDB code: 9BRA)^4^. Subunit A of V_1_ is colored yellow, and V_O_ is shown in black. The arrow points to the domain in subunit A that interacts with DMXL2. (**b**) Overlay of the cryo-EM structure with the AlphaFold3-predicted structure of DMXL2-V_1_ complex (see Fig. 4a). DMXL2 is shown in blue, subunit D of V_1_ in purple, and subunit B of V_1_ in red. **(c)** As in (**a**), but in a rotated view. **(d)** As in (**b**), but in a rotated view.

Our data support a model in which, under steady-state conditions, mRAVE interacts in the cytosol with subunits A and D of the inactive V_1_ complex (Fig. 6, stage 1). As shown by mutagenesis, these interactions are mediated by the helical bundle domain of DMXL1/2 and are necessary for mRAVE function. We propose that the C-terminal tail of subunit D is especially critical, as it may interact only in the inactive state—when D is not rotating—explaining why mRAVE binds inactive V_1_ but not the active V-ATPase holoenzyme, despite subunit A of V_1_ being accessible in both cases (Extended Data Fig. 8).

Following proton gradient dissipation, a fraction of mRAVE assembles into a supercomplex with V_1_ and V_O_ on lysosomal or Golgi membranes (Fig. 6, stage 2). This supercomplex consists of mRAVE, V_1_ and V_O_ and is probably an intermediate in which V_1_ and V_O_ are not yet associated. We speculate that in this supercomplex, DMXL1/2 is bound to V_1_ and WDR7 to V_O_. ROGDI serves as a linker, as also recently suggested by others from AlphaFold modeling^26^. The binding of WDR7 to V_O_ would be regulated and transient, as no interaction was detected in untreated cells. The dissociation of mRAVE from the supercomplex would enable the assembly of the V-ATPase holoenzyme (Fig. 6, stage 3) and the re-establishment of the proton gradient (Fig. 6, stage 4).

We also found that ATG16L1 is recruited to either preexisting or newly assembled V-ATPase complexes on monensin treatment and then initiates CASM. Thus, CASM seems to be a cellular response to a situation where organelles, such as lysosomes, have an intact V-ATPase but have lost their proton gradient (Fig. 6, stage 3). The exact downstream events of CASM remain unclear, although some insights have recently been provided^57–61^.

The collapse of proton gradients in organelles is probably the decisive signal that activates mRAVE, causing the subsequent V_1_–V_O_ assembly and ATG16L1 recruitment. Indeed, this assumption can explain why these processes can be triggered by diverse stimuli that all converge on proton leakage. These stimuli include agonists of the TRPML1 calcium–proton channel^61,62^, activation of the stimulator of interferon genes (STING) proton channel^63,64^, and proton ionophores, including monensin and nigericin^30^. Pathogens can also induce V_1_–V_O_ assembly through proton leakage. For example, influenza A infection triggers V_1_–V_O_ assembly through its M2 proton channel^32,35^.

How the collapse of the proton gradient is detected remains unclear, but a good candidate for a sensor is the V_O_ subcomplex itself. This hypothesis is supported by the observed effects of BafA1, a specific inhibitor of the V_O_ complex that binds reversibly to the transmembrane helices of the c ring^40^. While both BafA1 and monensin cause dissipation of proton gradients, only monensin, and not BafA1, promotes the formation of the supercomplex containing mRAVE, V_1_ and V_O_ (Fig. 5c). BafA1 prevents the monensin-induced assembly of V_1_ and V_O_ (Fig. 5h). These findings suggest that the dissipation of the proton gradient triggers a conformational change in V_O_, enabling mRAVE to recruit V_1_ and catalyze holoenzyme assembly. BafA1 may disrupt the conformational change, thereby inhibiting mRAVE from catalyzing assembly. Our data show that after washout of BafA1, mRAVE is required for the V-ATPase to return to its active conformation, although no change in the association of V_1_ with V_O_ is discernible. It remains unclear how, in this case, mRAVE reactivates the V-ATPase.

We and others observed that many V-ATPase molecules in untreated mammalian cells are dissociated into V_1_ and V_O_, indicating dynamic assembly–disassembly^38^ (Fig. 5f). This likely maintains optimal proton gradients in organelles, consistent with the rapid exchange of V_1_ between the cytosol and the lysosomal membrane^21^. When luminal pH rises, V_O_ may undergo a conformational change, allowing mRAVE-mediated holoenzyme assembly. Once the acidic pH is restored, V_O_ may revert, prompting V_1_ dissociation. Indeed, gradient restoration after monensin washout coincides with V_1_–V_O_ disassembly (Extended Data Fig. 7d), and structural data suggest V-ATPase disassembly occurs under conditions of restored acidity, such as in ATP-treated synaptic vesicles^5^. This model also explains previous findings that V-ATPase assembly in neurons is pH dependent^19^. How the V_O_ complex would sense the luminal proton concentration, how this information would be transmitted to mRAVE to initiate V_1_–V_O_ assembly and how ATG16L1 would be recruited to the V-ATPase holoenzyme when the proton gradient collapses all remain important questions to be answered.

Our findings also provide a molecular basis for neurological disorders linked to mutations in mRAVE subunits. Homozygous null mutations in DMXL2 are associated with Ohtahara syndrome, a progressive epileptic encephalopathy^65^, and homozygous null mutations in *ROGDI* cause Kohlschütter–Tönz syndrome, a condition marked by enamel formation defects, seizures, severe developmental delays and intellectual disability^24,66^. In individuals lacking ROGDI, mRAVE is partially functional, as our data show that in the absence of ROGDI the other subunits (DMXL1/2 and WDR7) can still weakly interact and retain much of their activity in lysosome acidification. Mutations in mRAVE likely reduce V-ATPase regulation, affecting critical processes such as organelle homeostasis, neurotransmission and extracellular space acidification. A small molecule that enhances the interaction between DMXL1/2 and WDR7 could potentially be used to treat Kohlschütter–Tönz syndrome.

## Methods

The research conducted for this study complies with Harvard Medical School’s ethical regulations and was approved by the Committee on Microbiological Safety.

### Cell culture

HEK-293T cells (ATCC, CRL-3216, RRID:CVCL_0063) were grown in Dulbecco’s modified Eagle’s medium (DMEM) (Thermo Fisher Scientific, catalog number (cat. no.) 11965118) supplemented with 100 units ml^−1^ penicillin, 0.1 mg ml^−1^ streptomycin (Thermo Fisher Scientific, cat. no. 15070063) and 10% fetal bovine serum (FBS) (Cytiva, cat. no. SH30088.03). The cells were maintained at 37 °C with 5% CO_2_. For the specified experiments, cells were exposed to 100 nM Bafilomycin A1 (Cell Signaling Technology, cat. no. 54645S) for 1 h, 100 µM monensin (Sigma, cat. no. M5273) for 1 h, 20 µM nigericin (Sigma, cat. no. N7143) for 2 h, 1 µM dTAG13 (MedChemExpress, cat. no. HY-114421) for 4 h, 10 µM MG132 (Selleckchem, cat. no. S2619) for 4 h and 100 ng ml^−1^ of doxycycline (Sigma, cat. no. D9891) unless indicated otherwise.

### Plasmids and cloning

The genome-wide CRISPR–Cas9 sgRNA Root library (five sgRNAs per gene, 94,335 sgRNAs total) was used previously^69,70^. The VMAT2 (SLC18A2) plasmid complementary DNA (cDNA), which includes a stop codon and confers kanamycin resistance, was obtained from the human Ultimate Open Reading Frame collection (Thermo Fisher Scientific) as a Gateway entry clone (IOH43207, NM_003054.3). This VMAT2 entry clone was then subcloned into a cytomegalovirus promoter destination vector that provides hygromycin resistance through a Gateway LR recombination reaction (Thermo Fisher Scientific, cat. no. 11791100).

Untagged versions of human DMXL1, DMXL2, WDR7 or ROGDI were inserted into the pRK5 vector. The pRK5 backbone (Addgene, no. 46328) was first digested with EcoRI and BamHI, and then fragments of DMXL1, DMXL2, WDR7 or ROGDI, containing pRK5-compatible overhangs, were synthesized by Integrated DNA Technologies (IDT). These fragments were ligated into the digested pRK5 vector using HiFi DNA Assembly (NEB, cat. no. E2621L). To introduce single Flag or HA tags or mutations to the respective plasmids, polymerase chain reaction was performed with the Q5 Site-Directed Mutagenesis kit (NEB, cat. no. E0554S), using primers designed via the NEBasechanger program.

For generating WDR7-dTAG-3xHA or DMXL2-dTAG-3xHA, the homology-directed repair (HDR) backbone plasmid (Addgene, 113097) was digested with HindIII and NotI. Three fragments from IDT, each containing 1,000 bp of genomic homology upstream or downstream of the stop codon, along with the dTAG-3xHA-P2A-Puromycin coding sequence, were ligated into the digested backbone using HiFi DNA Assembly. The genomic region targeted by the sgRNA was also modified to avoid cleavage after repair. To construct the lentiviral CRISPR–Cas9 vectors containing the desired sgRNA, the lentiCRISPRv2 backbone (Addgene, 52961) was digested with BsmBI. sgRNA oligos, which included CACC or AAAC overhangs, were synthesized by IDT, phosphorylated, annealed and ligated into the linearized backbone using T4 ligase (NEB, cat. no. M0202S).

sgRNA DMXL1 GCTAGCAAAGAACCATCCAT

sgRNA DMXL2 GTGACATTCTATAAAGATTG

sgRNA WDR7 GGCAGCATTAGACCATGAAG

dTAG-3xHA-P2A-Puro sequence:

GGAGGCGGTTCAGGGGTACAGGTCGAGACAATAAGTCCGGGGGACGGTAGAACTTTCCCCAAAAGGGGCCAGACGTGTGTTGTGCATTATACGGGCATGTTGGAGGATGGAAAGAAAGTAGACTCCTCACGGGATAGAAACAAACCCTTTAAATTTATGTTGGGGAAGCAAGAGGTTATTAGGGGATGGGAAGAGGGTGTAGCCCAGATGTCTGTAGGCCAACGCGCAAAATTGACTATAAGTCCAGATTATGCGTACGGCGCTACCGGTCACCCTGGGATAATTCCCCCACACGCCACGCTGGTGTTTGATGTCGAACTTCTCAAACTGGAAGGCTCTGGTTATCCGTACGATGTACCTGATTATGCCGGGTATCCCTACGACGTGCCAGACTATGCTGGTTATCCGTATGACGTTCCGGACTATGCTGGTGCGACCAATTTTAGTTTGTTGAAACAGGCGGGTGATGTGGAGGAAAACCCGGGGCCTATGACCGAGTATAAACCCACCGTAAGACTGGCGACCAGGGACGACGTTCCAAGGGCTGTCCGGACGCTGGCTGCTGCGTTCGCGGATTACCCTGCCACTAGGCACACGGTTGACCCCGATCGACATATTGAAAGGGTAACTGAACTTCAAGAGTTGTTCCTGACGCGGGTAGGTCTGGATATAGGAAAAGTATGGGTAGCTGATGATGGCGCGGCTGTGGCAGTCTGGACCACTCCGGAAAGCGTTGAAGCTGGCGCAGTTTTCGCTGAAATCGGTCCCAGGATGGCAGAACTTAGTGGGTCCCGATTGGCGGCACAACAGCAAATGGAGGGACTGCTTGCACCGCACCGGCCTAAAGAGCCCGCGTGGTTCCTTGCCACCGTAGGTGTATCTCCTGATCACCAGGGAAAAGGTCTTGGCTCCGCCGTTGTGCTCCCGGGTGTTGAGGCTGCCGAAAGAGCAGGAGTCCCTGCATTTTTGGAAACTAGTGCCCCGCGAAACCTCCCTTTTTACGAACGCTTGGGTTTCACAGTCACGGCCGACGTTGAAGTCCCAGAGGGACCCAGAACGTGGTGCATGACCAGGAAACCCGGGGCT

Helical bundle domain amino acid sequence:

MYPYDVPDYAGGGSGQEHARVLSSHLMHSSLPGLTRLEQMFLVALADTVATTSTELDESRDKSCSGRDTLDECGLRYLLAMRLHTCLLTSLPPLYRVQLLHQGVSTCHFAWAFHSEAEEELINMIPAIQRGDPQWSELRAMGIGWWVRNINTLRRCIEKVAKASFQRNNDALDAALFYLSMKKKAVVWGLFRSQHDEKMTTFFSHNFNEDRWRKAALKNAFSLLGKQRFEQSAAFFLLAGSLKDAIEVCLEKMEDIQLAMVIARLYESEFETSSTYISILNQKILGCQKDGSGFSCKRLHPDPFLRSLAYWVMKDYTRALDTLLEQTPKEDDEHQVIIKSCNPVAFSFYNYLRTHPLLIRRNLASPEGTLATLGLKTEKNFVDKINLIERKLFFTTANAHFKVGCPVLALEVLSKI

HA-DMXL2 WT or mutants were PCR amplified from the pRK5 vector. A Sleeping Beauty plasmid^71^ containing blasticidin resistance was digested with SfiI, and HA-DMXL2 was inserted via HiFi DNA Assembly.

### Generating genomically edited cell lines

#### Generation of DMXL1-knockout HEK-293T cells

WT HEK-293T cells were transfected with the lentiCRISPRv2 blue fluorescent protein (BFP) plasmid containing a sgRNA targeting exon 11 of DMXL1. Five days posttransfection, BFP-positive cells were single-cell sorted into 96-well plates. Clones were expanded, and an aliquot of 200,000–400,000 cells from each clone was collected by centrifugation. Genomic DNA for genotyping was extracted by resuspending the cell pellet in 100 µl of 50 mM NaOH, vortexing and heating at 95 °C for 1 h. After cooling, the mixture was vortexed and centrifuged, and 50 µl of 1 M Tris (pH 7.4) with 4 mM EDTA was added to neutralize the solution. Following another vortexing and centrifugation at 21,000*g* for 5 min, the supernatant containing genomic DNA was collected. For PCR amplification of the sgRNA target site, 35 cycles of PCR were performed (annealing at 54.5 °C) using DreamTaq (Thermo Fisher, cat. no. K1081) and the following primers:

Forward: GTAATAACCTGTTTGTATTTACTTGG

Reverse: GTACAGGCATACATCATTATGC

PCR products were gel-purified and sequenced by the Sanger technique using the following primers:

CCGAAAAGAAGGAATTAGGC

GGTTCATGAACTCTTTTGC

GAGAGAGTTTGCATCACC

Mutation and allele analysis was performed using Synthego’s ICE CRISPR Analysis Tool.

#### Generation of WDR7-dTAG-3xHA-P2A-Puro cell lines

WT HEK-293T cells were cotransfected with the WDR7-dTAG-3xHA-P2A-Puro HDR plasmid and the lentiCRISPRv2 puromycin plasmid containing a sgRNA targeting the C-terminal region of WDR7. Two days posttransfection, cells were selected with puromycin and cultured for 2 weeks, passaging as needed. Puromycin-resistant cells were then single-cell sorted into 96-well plates, and clones were expanded. An aliquot of 200,000–400,000 cells from each clone was gathered by centrifugation, and genomic DNA was extracted as described above. PCR was performed using 35 cycles (annealing at 54.5 °C) with the following primers flanking the WDR7 C terminus to check for heterozygous or homozygous insertion:

Forward: CTGCATTAAAACCTACCAGG

Reverse: GAAAAACTACCTGGTGACAG

Further sequencing and immunoblotting confirmed successful biallelic, in-frame insertion.

#### Generation of DMXL2-dTAG-3xHA-P2A-Puro cell lines

*DMXL1*-KO HEK-293T cells were cotransfected with the DMXL2-dTAG-3xHA-P2A-Puro HDR plasmid and the lentiCRISPRv2 puromycin plasmid expressing a sgRNA targeting the C-terminal region of *DMXL2*. The remainder of the procedure followed the same steps as the WDR7-dTAG-3xHA-P2A-Puro cell line generation. PCR was performed with 35 cycles (annealing at 57 °C) using the following primers flanking the *DMXL2* C-terminus to identify heterozygous or homozygous insertion:

Forward: GTTTAGATAATACTCAGCTTGGGTC

Reverse: TCATGCAGATTGACATCATCC

Successful biallelic, in-frame insertion was confirmed by sequencing and immunoblotting.

### Generating cell lines stably overexpressing HA-DMXL2

An isogenic clone containing *DMXL1-*KO and endogenous DMXL2-dTAG-3xHA was seeded into six-well plates at a density of 200,000 cells per well and allowed to grow for 2 days. The cells were then transfected using PolyJet (SignaGen, cat. no. SL100688) following the manufacturer’s instructions using 950 ng of Sleeping Beauty plasmid HA-DMXL2 and 50 ng of transposase plasmid^71^. Two days posttransfection, the cells were expanded into 15-cm plates in the presence of blasticidin. The cells were cultured under blasticidin selection for 2–3 weeks before being used for downstream assays.

### Lentivirus production

HEK-293T cells were seeded into six-well plates at a density of 300,000 cells per well and allowed to grow for 2 days. They were then transfected using PolyJet (SignaGen, cat. no. SL100688) following the manufacturer’s instructions. In short, plasmids encoding Tat, Rev, Gag-Pol and VSV-G were mixed in equimolar with a lentiviral transfer vector. A total of 1 µg of plasmid DNA was diluted in un-supplemented DMEM, and 3 µl of PolyJet reagent was separately diluted in the same medium. The PolyJet reagent was then combined with the DNA solution, incubated for 15 m at room temperature, and added dropwise to the cells. One day after transfection, the medium was replaced with fresh medium. Lentiviral supernatant was harvested 48 h posttransfection, filtered through a 0.45-µm filter and directly applied to the cells. For library preparation, lentivirus was produced by transfecting 13 µg of total DNA per 15-cm plate across eight 15-cm plates. The virus was then pooled, concentrated with the lenti-X concentrator (Takara, cat. no. 631232), divided into aliquots and frozen at −80 °C.

### Genome-wide CRISPR–Cas9 screen

The genome-wide screen was carried out as previously described^70,72^. The CRISPR Root plasmid library was packaged into lentivirus by transfecting HEK-293T cells with PolyJet, as described earlier. The lentivirus was titered to achieve a multiplicity of infection of approximately 0.3. HEK-293T cells were then transduced with the titered CRISPR–Cas9 genome-wide Root library lentivirus at an multiplicity of infection of ~0.3 to ensure a 500× representation. After 48 h, cells were selected for 4 days with puromycin (2 µg ml^−1^) to eliminate uninfected cells. On day 6 posttransduction, the cells were treated with 100 nM BafA1 for 1 h, rinsed twice with fresh medium lacking BafA1 followed by a 2-h recovery period in fresh medium and then stained with 1 µM LysoTracker (Thermo Fisher Scientific, L7528) for 1 h. The cells were washed once with PBS, detached with trypsin and the 95th percentile lowest Y675-PC5-A (far red) cell population was sorted using fluorescence-activated cell sorting with a MoFlo Astrios instrument (Beckman Coulter). Additionally, the unsorted input cells were collected in parallel. The collected cells were washed with PBS, pelleted and stored at −80 °C.

Once thawed, the cell pellets were used to extract genomic DNA with a Gentra Puregene Core Kit from Qiagen. The sgRNAs were then amplified via PCR using Q5 Hot Start High-Fidelity DNA Polymerase from NEB and 4 µg of genomic DNA as the template per reaction (all genomic DNA was used), incorporating stagger sequences. A second round of PCR was conducted with the purified PCR1 product to add Illumina P5 and P7 adapters. The PCR2 products were cleaned, pooled in the correct ratios and sequenced on a NextSeq 500 instrument. The raw sequencing data was processed using Cutadapt^73^ to extract sgRNAs and then mapped to the reference library using Bowtie2 (ref. ^74^). MAGeCK^67^ was used to analyze the enrichment of sgRNAs in the 95th percentile compared to the input population. The MAGeCK score, plotted on the *y* axis, represents the negative log_10_ of the ‘pos|score’ value generated by MAGeCK.

### pH recovery, neurotransmitter loading and flow cytometry

HEK-293T cells were plated in six-well plates and 2 days later pretreated with 1 µM dTAG13 for 4 h. The cells were then treated with 100 nM BafA1 for 1 h in the presence of dTAG13. After treatment, the medium was discarded and the cells were washed twice with fresh medium. After the second wash, fresh medium containing dTAG13 was added and the cells were incubated for 2 h at 37 °C to recover lysosomal acidification. The medium was then removed, and the cells were stained with 1 µM LysoTracker (Thermo Fisher Scientific, cat. no. L7528) for 1 h at 37 °C. Following the stain, the cells were rinsed once with PBS, detached using 0.05% trypsin and the trypsin was quenched with fresh medium.

For neurotransmitter loading, HEK-293T cells were first infected with lentivirus to stably express VMAT2 and then selected with blasticidin. The same recovery procedure was followed, but instead of using LysoTracker, the cells were stained with 5 µM FFN206 (Tocris, cat. no. 5043) for 2 h. Approximately 10,000 individual cells were analyzed for YG602-A (LysoTracker) or V450-A (FFN206) using a CytoFLEX S flow cytometer. Flow cytometry data were collected using CytExpert software (Beckman Coulter), and figures were created using the FlowJo software.

### siRNA transfection

HEK-293T cells were plated at 175,000 cells per well in a six-well plate and incubated overnight. The next day, the medium was replaced 1 h before transfection with 1.5 ml of fresh medium containing FBS and antibiotics. Small interfering RNAs (siRNAs) were reconstituted in 1× siRNA buffer (Horizon, cat. no. B-002000-UB-100) to create a 20 μM stock solution. The following siRNAs were used:

Nontargeting Control (Horizon, cat. no. D-001810-01-05)

WDR7 (Horizon, cat. no. LQ-012867-00-0002)

ROGDI (Horizon, cat. no. LQ-014379-02-0002)

For transfection, 6 μl of the siRNA (20 μM) was diluted in 250 μl of Opti-MEM medium (Thermo Fisher Scientific, 31,985,070) without FBS or antibiotics, and the mixture was vortexed briefly. Then, 5 μl of Lipofectamine RNAiMAX (Thermo Fisher Scientific, 13,778,075) was diluted in 250 μl of Opti-MEM medium without FBS or antibiotics. The RNAiMAX solution was mixed with the siRNA solution to form a total of 500 μl of the siRNA–RNAiMAX complex. After vortexing briefly, the mixture was incubated at room temperature for 18 min to allow complex formation. The entire 500 μl of solution was added to the cells, ensuring a final siRNA concentration of 60 nM, and the cells were incubated overnight. The medium was then replaced the following day, and gene expression was assessed by immunoblotting approximately 48–72 h posttransfection. The lysosomal reacidification assay was carried out 48–72 h posttransfection.

### Purification of mRAVE

A total of 3.5 million HEK-293T cells were plated in each of 30 15-cm dishes. Two days later, the cells were transfected using PolyJet, with 8 µg of DNA per dish: 5,982 ng of pRK5 DMXL2, 1,834 ng of WDR7 and 185 ng of ROGDI-Flag. This followed an optimized molar ratio of 13:6:1, with 500 µl of DMEM + DNA and 500 µl of DMEM + 30 µl of PolyJet per dish. The medium was replaced 1 day after transfection. Two days posttransfection, cells were washed with ice-cold PBS and gathered by scraping in 1 ml of lysis buffer containing 50 mM HEPES, 150 mM NaCl, 1× protease inhibitors (Selleckchem, cat. no. B14002) and 1% Triton-X-100 per dish. The lysate was incubated with end-to-end rotation for 30 min at 4 °C and then centrifuged at 21,000*g* for 20 min at 4 °C. The clarified lysate was transferred into two 50-ml Falcon tubes. M2 Flag agarose resin (Millipore, cat. no. A2220, RRID:AB_10063035, 1.8 ml of packed resin, prewashed four times with lysis buffer) was added to the lysate and the mixture was rotated for 1.5 h at 4 °C. The Falcon tubes were sealed with parafilm to prevent leakage. After incubation, the resin–lysate mix was passed through a column (BioRad, 7321010) at 4 °C by gravity. The Falcon tubes were rinsed with 15 ml of lysis buffer each, and the rinse was passed through the column. The resin on the column was then washed with 20 ml of lysis buffer, followed by 20 ml of SEC buffer (50 mM HEPES, 150 mM NaCl, 0.05% CHAPS). Proteins were eluted using SEC buffer containing 0.5 mg ml^−1^ 3xFlag peptide (APExBIO, A6001), incubated for 18 min at room temperature and the eluate was collected by gravity flow. This elution was repeated for a total of four times. To check for the presence of proteins, absorbance was measured at 280 nm, using the elution buffer as the blank. Eluates containing proteins were pooled and concentrated to about 1 ml using an Amicon Ultra-15 50-kDa molecular weight cutoff at 3,500*g*. It was essential to prewet the filter with SEC buffer and pipette gently to avoid precipitating the protein. After concentrating, the protein was centrifuged at 21,000*g* for 1 min at 4 °C to remove aggregates. A Superose6 column was equilibrated with SEC buffer, and a 1-ml injection loop was washed with SEC buffer. Next, 1 ml of the concentrated protein was injected into the column. The 0.35-ml fractions corresponding to the protein peaks (main and shoulder) were collected, pooled and concentrated further using an Amicon Ultra-2 50-kDa molecular weight cutoff at 4,800*g*. The final volume was approximately 300 µl, at a concentration of 3–4 mg ml^−1^ of pure protein. The protein was flash-frozen and stored at −80 °C. The unconcentrated purified proteins from the peak fraction were used directly for negative-stain electron microscopy.

### Negative-stain electron microscopy and model generation

Purified mRAVE was diluted to a concentration of 0.02 mg ml^−1^ and 3.5 µl of this diluted protein was applied to glow-discharged 400-mesh copper grids (EMS400-Cu) coated with a continuous 10-nm carbon layer. The grids were left for 1 min before being blotted with filter paper (VMR, cat. no. 28310-081). They were then stained for a few seconds with a 1.5% uranyl formate solution (Electron Microscopy Science, cat. no. 22451) and blotted again to remove excess liquid. This staining procedure was repeated twice, followed by an additional 30-s incubation in uranyl formate and another blotting step. The grids were then allowed to air-dry at room temperature before imaging. In total 30 images were captured using a FEI Tecnai T12 transmission electron microscope operating at 120 kV, with a Gatan UltraScan 895 (4,000 × 4,000) CCD camera at a nominal magnification of ×52,000, which corresponds to a pixel size of 2.13 Å and a defocus setting of −1.5 to −3.0 µm. The images were processed using RELION v.4.0.1 (ref. ^75^). Specifically, particles were autopicked using a Laplacian-of-Gaussian-based auto-picking method (minimum diameter 200 Å, maximum diameter 220 Å and ‘Are the particles white?’ Yes) and then extracted using the particle box size of 100 pixels as well as no invert contrast. A total of 27,727 particles underwent multiple 2D classifications to remove junk particles. 2D classes based on ~12,000 particles were used as templates for particle picking. The resulting 37,063 particles were extracted using the same particle box size and then subjected to 2D classification (*N* = 100, *T* = 2, mask diameter 200 Å). After multiple rounds of 2D classification, a clean dataset containing 23,531 particles was used to generate an initial three-dimensional (3D) classification (*N* = 2, *T* = 4, mask diameter 200 Å). All other parameters were set to default settings.

### Coomassie blue staining

Tris-Glycine gels were soaked in Coomassie Brilliant Blue R-250 staining solution (BioRad, cat. no. 1610436), microwaved for 15 s and then incubated at room temperature with gentle rocking for 30 min. The gels were subsequently destained overnight by gently rocking in a solution of 35% methanol and 10% acetic acid in water, with a Kimwipe placed in the solution.

### Purification of recombinant SidK-Flag

Here, 20 µl of chloramphenicol-resistant Rosetta (DE3) cells (Millipore, cat. no. 71397-3) were transformed with a plasmid that confers kanamycin resistance and isopropyl-β-d-thiogalactoside (IPTG)-inducible expression of 6xHis-SidK-Flag (Addgene, no. 175787). The transformed cells were plated on chloramphenicol- and kanamycin-containing agar plates and incubated at 37 °C overnight. The following day, a single colony was picked and transferred into 50 ml of Luria-Bertani starter cultures with the appropriate antibiotics, then grown overnight at 37 °C with shaking at 200 rpm. The next morning, these starter cultures were transferred into 1 l of fresh Luria-Bertani with antibiotics and grown at 37 °C until an optical density of 0.6 was reached. At this point, the culture was cooled at 4 °C for 10 min, and 0.5 mM IPTG (Sigma, cat. no. I5502-10G) was added to induce protein expression. The cultures were then grown at 16 °C for 16–18 h.

After induction, the bacteria were harvested by centrifugation at 4,000*g* for 15 min at 4 °C. The pellet was resuspended in 35 ml of buffer containing 50 mM HEPES pH 7.4, 150 mM NaCl and 10 mM imidazole (Sigma, cat. no. 68268-500ML-F), and then pelleted again at 4,000*g* for 15 min at 4 °C. The supernatant was discarded, and the pellet was resuspended in 35 ml of lysis buffer containing 50 mM HEPES pH 7.4, 150 mM NaCl, 10 mM imidazole, 1 mM dithiothreitol (Thermo Fisher Scientific, cat. no. R0862), benzonase (Millipore, cat. no. 71206-3) and protease inhibitor tablets (Sigma, cat. no. 11873580001). Cells were then lysed using a microfluidizer at 700 bar for four cycles with a 20 ml min^−1^ flow rate. The lysate was clarified by centrifugation at 21,000*g* for 20 min at 4 °C. During this process, an 0.8-ml column volume (1.6 ml in total for the protein preparation) of Ni-NTA agarose (Qiagen, cat. no. 30210) was washed three times with lysis buffer. The clarified supernatant was then incubated with the washed Ni-NTA agarose in a 50-ml conical tube for 1 h with end-to-end rotation at 4 °C. After incubation, the mixture was passed through a column by gravity flow, and both the tube and column were washed with 50–60 ml of buffer at 4 °C. Protein was eluted from the resin four times, with 500 µl of buffer containing 50 mM HEPES, 150 mM NaCl and 200 mM imidazole for 5 min per elution. The absorbance at 280 nm was measured for each elution using a Nanodrop apparatus. The protein elutions were pooled, flash-frozen in aliquots and stored at −80 °C.

### Immunoprecipitation

HEK-293T cells were plated at a density of 0.9 million cells per 10-cm culture dish. Two days after plating, the cells were transfected with a total of 3 µg of the specified plasmid(s) using PolyJet. The medium was replaced with fresh medium 1 day after transfection. Two days posttransfection, the cells were washed once with ice-cold PBS and 700 µl of lysis buffer containing 50 mM HEPES, 150 mM NaCl, 1× Halt protease inhibitors and 1% Triton-X-100 was added per dish. For IPs of endogenous mRAVE subunits, the cells were grown to approximately 40 million cells per 15-cm dish before lysing. The cells were gathered by scraping into Eppendorf tubes, and the lysates were incubated with end-to-end rotation for 30 min at 4 °C. The samples were then centrifuged at 21,000*g* for 20 min at 4 °C. During centrifugation, 15 µl of anti-Flag (Sigma, cat. no. M8823, RRID:AB_2637089) or anti-HA (Thermo Fisher Scientific, cat. no. 88836, RRID:AB_2749815) magnetic beads per plate were washed three times with 700 µl of lysis buffer. After centrifugation, 50 µl of the whole-cell lysate was collected and added to 150 µl of Tris-Glycine SDS sample buffer (Thermo Fisher Scientific, cat. no. LC2676) containing 10% 2-mercaptoethanol. The remaining supernatant was incubated with the magnetic beads for 1.5 h at 4 °C.

For immunoprecipitating the V_1_ subcomplex, cells grown to about 85% confluence in 10-cm dishes were lysed as described earlier, using 0.7 ml of buffer containing 50 mM HEPES, 150 mM NaCl, 1× Halt protease inhibitors and 1% *n*-dodecyl-β-d-maltoside. After collecting the lysate supernatant in a separate tube, approximately 35 µg of recombinant SidK-Flag protein was added to the lysate, and an aliquot of the input lysate containing SidK-Flag was taken. Anti-Flag magnetic beads were then added, and the mixture was incubated end-to-end for 1.5 h at 4 °C.

The beads were washed three times with 700 µl of lysis buffer and resuspended in 50 µl of Tris-Glycine SDS sample buffer. The protein samples were denatured by heating at 95 °C for 3 min. The immunoprecipitated samples were diluted 1:20 in Tris-Glycine SDS sample buffer for blotting the bait protein, and 15 µl of the immunoprecipitated sample or whole-cell lysate (5–10% of the immunoprecipitate, assuming 100% bait capture) was loaded to detect coimmunoprecipitated proteins by immunoblotting.

### Turbo-LysoIP

WDR7-dTAG-3xHA cells were transduced with lentivirus to stably express LAMP1-GFP-TurboID^54^. Cells were grown in four 15-cm plates until approximately 90% confluent. The cells were then pretreated with or without 1 µM dTAG13 for 4 h, followed by treatment with or without monensin for 30 min. After the first 30 min of monensin treatment, 50 µM biotin was added directly to the culture medium and the cells were incubated for an additional 30 min to allow biotinylation of lysosomal surface proteins. The cells were washed twice with ice-cold PBS and collected by scraping with 1 ml of lysis buffer containing 50 mM HEPES, 150 mM NaCl, 1× Halt protease inhibitors and 1% Triton-X-100.

The lysates were incubated with end-to-end rotation for 30 min at 4 °C and then centrifuged at 21,000*g* for 20 min at 4 °C. During centrifugation, 30 µl of Pierce Streptavidin magnetic beads (Thermo Fisher Scientific, cat. no. 88815) per plate were washed three times with 1 ml of lysis buffer. After centrifugation, 50 µl of the whole-cell lysate was collected and added to 150 µl of Tris-Glycine SDS sample buffer containing 10% 2-mercaptoethanol. The remaining supernatant was incubated with the magnetic beads for 1.5 h at 4 °C. The beads were washed three times with 1 ml of lysis buffer and resuspended in 50 µl of Tris-Glycine SDS sample buffer. Protein samples were then denatured by heating at 95 °C for 3 min. Finally, 15 µl of whole-cell lysate or IP sample was loaded into gels for protein detection by immunoblotting.

### Crude cell fractionation

HEK-293T cells expressing WDR7-dTAG-HA from the endogenous locus were cultured to about 80% confluency in two 15-cm dishes. The cells were then treated with and without 100 µM monensin for 1 h. The cells were washed once with ice-cold PBS and collected by scraping with 1 ml of ice-cold PBS. The cells were lysed by dounce homogenizing 50 times. The cell lysates were centrifuged at 1,500*g* for 4 min at 4 °C to remove unbroken cells and/or nuclei. A 50-µl aliquot of each supernatant was diluted into 50 µl of Tris-Glycine SDS sample buffer containing 10% 2-mercaptoethanol (input). The remainder of the supernatants were ultracentrifuged for 1 h at 100,000*g* and 4 °C. The resulting supernatants were removed, and a 50-µl aliquot of each was diluted into 50 µl of sample buffer (soluble). The pellets were rinsed with 1 ml of cold PBS and ultracentrifuged again for 30 min. The resulting pellets were resuspended in 2 ml of sample buffer (insoluble). Samples were then denatured by heating at 95 °C for 3 min. Finally 15 µl of each sample was loaded into gels for protein detection by immunoblotting.

### Immunoblotting

Protein samples were resuspended in Tris-Glycine SDS sample buffer (Thermo Fisher Scientific, cat. no. LC2676) containing 10% 2-mercaptoethanol and heated at 95 °C for 3 min to denature the proteins. The denatured samples were then loaded into 4–12% or 4–20% (for detecting LC3) Tris-Glycine 15-well precast gels (Thermo Fisher Scientific, cat. nos. XP04125BOX or XP04205BOX), and electrophoresis was performed at a constant 165 volts in 1× Tris-Glycine SDS running buffer until the molecular weight marker (Thermo Fisher Scientific, cat. no. 26619) reached the bottom of the gel. Proteins were then transferred to a 0.2-µm nitrocellulose membrane (BioRad, cat. no. 170-4158) using the Trans-Blot Turbo Transfer System (BioRad). The membranes were blocked with 5% milk (LabScientific, cat. no. M-0842) in 1× tris-buffered saline with Tween (TBST) for 15–30 min with gentle rocking at room temperature.

All primary antibodies were diluted to 1:1,000 by volume in the blocking solution and incubated overnight with gentle rocking at 4 °C. The following primary antibodies were used: rabbit anti-Flag (Cell Signaling Technology, 14793, RRID:AB_2572291), anti-Actin (Cell Signaling Technology, 4970, RRID:AB_2223172), rabbit anti-HA (Cell Signaling Technology, 3724, RRID:AB_1549585), rabbit anti-mTOR (Cell Signaling Technology, 2983, RRID:AB_2105622), rabbit anti-ROGDI (Proteintech, 17047-1-AP, RRID:AB_2182328), rabbit anti-DMXL2 (Abcam, ab234771, RRID:AB_3675331), rabbit anti-ATP6V1A (Proteintech, 17115-1-AP, RRID:AB_2290195), rabbit anti-ATP6V1B2 (Cell Signaling Technology, 14617, RRID:AB_2798541), rabbit anti-ATP6V1C1 (Proteintech, 16054-1-AP, RRID:AB_2062501), rabbit anti-ATP6V1D (Proteintech, 14920-1-AP, RRID:AB_2243302), rabbit anti-ATP6V1E1 (Proteintech, 15280-1-AP, RRID:AB_2062545), rabbit anti-ATP6V1F (Proteintech, 17725-1-AP, RRID:AB_2062680), rabbit anti-ATP6V1G2 (Proteintech, 25316-1-AP, RRID:AB_2880027), rabbit anti-ATP6V1H (Proteintech, 26683-1-AP, RRID:AB_2880601), rabbit anti-ATP6V0A1 (Proteintech, 13828-1-AP, RRID:AB_2877979), rabbit anti-ATP6V0A2 (Abcam, ab96803, RRID:AB_10680914), rabbit anti-SKP1 (Cell Signaling Technology, 2156, RRID:AB_2270271), rabbit anti-ATG16L1 (Cell Signaling Technology, 8089, RRID:AB_10950320), rabbit anti-LC3 (Cosmo Bio, MBL-PM036, RRID:AB_2274121), rabbit anti-PSMD4 (CST, 12441, RRID:AB_2797916) and mouse anti-LAMP2 (Santa Cruz Biotechnology, sc-18822, RRID:AB_626858).

After incubating overnight with the primary antibody, the membranes were washed three times with 1× TBST for quick rinses, followed by three 5-min washes with gentle rocking in 1× TBST. Following the rinses, the membranes were incubated with 5% milk in 1× TBST containing a 1:2,000 dilution by volume of anti-rabbit IgG, horseradish peroxidase (HRP)-linked secondary antibody (Cell Signaling Technology, cat. no. 7074, RRID:AB_2099233) or anti-mouse IgG, HRP-linked secondary antibody (Cell Signaling Technology, 7076, RRID:AB_330924). The membranes were incubated with the secondary antibody for 1 h at room temperature with gentle rocking, then washed in the same manner as after the primary antibody incubation. After washing, the membranes were treated with either Pierce ECL western blotting substrate (Thermo Fisher Scientific, cat. no. 32106) for strong antibodies or abundant proteins, or Immobilon western chemiluminescent HRP substrate (Sigma, cat. no. WBKLS0500) for weaker antibodies or less abundant proteins for 1–2 min at room temperature. High-sensitivity autoradiography film (Denville Scientific, cat. no. E3218) was used to capture the immunoblotting results.

### Mass spectrometry of main and shoulder peaks

Here 50 µg of purified main peak or shoulder peak proteins (*n* = 1, per biological sample) were diluted to 100 µl in 50 mM Tris (pH 8.5) with 5% SDS. The proteins were denatured by heating at 95 °C for 3 min. The proteins were then reduced by incubating with 5 mM tris(2-carboxyethyl)phosphine hydrochloride at 55 °C for 15 min, followed by alkylation with 20 mM iodoacetamide at room temperature for 30 min in the dark. Afterward, the proteins were acidified to a final concentration of 2.5% (v/v) using phosphoric acid. The solution was then diluted tenfold with 100 mM Tris (pH 7.55) in a 90% methanol to 10% water mixture. The sample was passed through an S-Trap column (Protifi, C02-micro-10) by multiple rounds of centrifugation at 4,000*g* for 30 s. The trapped protein was washed three times with 150 µl of 100 mM Tris (pH 7.55) in 90% methanol to 10% water, followed by a dry spin at 4,000*g* for 1 min. A 20-µl solution containing 50 mM ammonium bicarbonate (pH 8) and 2 µg of trypsin (Promega, bV5113) was added to each column. Digestion was carried out overnight at 37 °C in a humid chamber.

The peptides were eluted by three sequential 1-min centrifugations at 4,000*g*, using 40 µl of ammonium bicarbonate (pH 8) for the first, 40 µl of 0.2% formic acid in water for the second and 40 µl of 50% acetonitrile in water for the third. The pooled eluate was dried using a SpeedVac under reduced pressure, and the peptides were resuspended in 30 µl of 0.1% formic acid in water before liquid chromatography with tandem mass spectrometry analysis as previously described^70^.

A Human UniProt SwissProt proteome (downloaded July 2024) was used as the reference database for protein identification. The data was searched using FragPipe (v.18.0) and the MSFragger search engine. Tryptic peptides with up to two missed cleavages were considered. Cysteine carbamidomethylation was set as a fixed modification, while methionine oxidation was a variable modification, with up to four variable modifications allowed per peptide. The mass tolerances were 10 ppm for precursor ions and 0.04 Da for product ions. PeptideProphet was used to filter peptide hits, achieving a false discovery rate of 1%.

### Crosslinking mass spectrometry of main and shoulder peaks

Freshly prepared samples were crosslinked as previously described (*n* = 1, per biological sample)^76^. Briefly, amine-amine crosslinking reaction was performed with 2 mM BS3 (Thermo Fisher Scientific) in 100 mM HEPES, 100 mM NaCl, pH 7.8 for 1 h at room temperature. The extended-EDC crosslinking reaction was carried out for 1 h at room temperature in 100 mM MOPS (piperazine-*N*,*N*′-bis(2-ethanesulfonic acid) buffer, pH 7.0, containing 100 mM NaCl, 75 mM EDC, 16 mM sulfo-*N*-hydroxysuccinimide and 25 mM 2,2′-(ethylenedioxy)bis(ethylamine) (EDDA) (SIGMA-Aldrich). Reactions were quenched with hydroxylamine to a final concentration of 50 mM. All samples were reduced for 1 h in 2% SDS and 10 mM tris(2-carboxyethyl)phosphine, alkylated with 20 mM iodoacetamide in the dark for 1 h and quenched with 20 mM beta-mercaptoethanol for 15 min. Samples were then processed using the SP3 method^77^ and digested with LysC(Wako) for 3 h and then trypsin (Promega) for 6 h, both at a 1:25 enzyme:substrate ratio and 37 °C. Digested peptides were acidified with 10% TFA to pH ~2, desalted using stage tips with Empore C18 SPE Extraction Disks (3M) and dried under vacuum.

Samples were reconstituted in 5% formic acid and 5% acetonitrile and analyzed in the Orbitrap Eclipse Mass Spectrometer (Thermo Fisher Scientific) coupled to an EASY-nLC 1200 (Thermo Fisher Scientific) ultrahigh-pressure liquid chromatography pump, as well as a high-field asymmetric waveform ion mobility spectrometry interface. Peptides were separated on an in-house pulled 100-μm inner diameter column packed with 35 cm of Accucore C18 resin (2.6 μm, 150 Å, Thermo Fisher Scientific), using a gradient consisting of 5–35% acetonitrile (0.125% formic acid) over 180 min at roughly 550 nl min^−1^. The instrument was operated in data-dependent mode. Fourier transform mass spectrometry level 1 spectra were collected at a resolution of 60 K, with an automatic gain control target of 100% and a maximum injection time of 50 ms. The most intense ions were selected for tandem mass spectrometry for 1.5 s in top-speed mode, while switching among three field asymmetric ion mobility spectrometry compensation voltages, −40, −60 and −80 V, in the same method. Precursors were filtered according to charge state (allowed 3 ≤ *z* ≤ 7) and monoisotopic peak assignment was turned on. Previously interrogated precursors were excluded using a dynamic exclusion window (120 s ± 10 ppm). MS2 precursors were isolated with a quadrupole mass filter set to a width of 1.1 *m/z* and analyzed by Fourier transform mass spectrometry level 2, with the Orbitrap operating at 30,000 resolution, a nonlinear automatic gain control target of 250% and a maximum injection time of 150 ms. Precursors were then fragmented by high-energy collision dissociation at 30% normalized collision energy.

Mass spectra were processed and searched using the PIXL search engine^76^. The sequence database contained 1,845 proteins identified at 1% false discovery range in a noncrosslinked Comet^78^ search. For PIXL search, precursor tolerance was set to 15 ppm and fragment ion tolerance to 10 ppm. Methionine oxidation and protein N-terminal acetylation were set as variable modifications in addition to mono-linked mass of +156.0786 for BS3 and +130.110613 for EDDA. Crosslinker mass shift of +138.0681 was used for BS3, +112.100048 for EDDA and the zero-length crosslinker resulted in a loss of water (−18.010565) for E-EDC search. Based on preliminary searches, phosphorylation modifications were allowed (+79.96633041) on the three mRAVE proteins. The top 150 most abundant proteins by total spectral counts were considered by PIXL for crosslinking for BS3 and the top 80 for E-EDC. Matches were filtered to a 1% false discovery range on the unique peptide level using linear discriminant features as previously described^76^.

### AlphaFold3 modeling

The amino acid sequence of the indicated protein(s) was extracted from UniProt and folded using the AlphaFold3 web server^51^. Figures 2f, 3b and 4a and b and Extended Data Figs. 4a, 5a–d, 6a–c and 8a–d were made using ChimeraX v.1.4–1.7.

### Statistics and reproducibility

In flow cytometry experiments, median fluorescence intensity was measured using FlowJo version 10.7.1. For immunoblot analysis, band intensities were quantified with ImageJ. A uniform rectangular selection was applied to each lane to measure band intensity, and the same dimensions were maintained across all bands on a given gel. A similarly sized box was placed over a blank area to assess background intensity. The band signal was determined by subtracting the background intensity from the total intensity. Lipidated LC3 band intensities were normalized to actin levels (loading control) by dividing the LC3 intensity by that of actin. These ratios were then normalized to the value at the zero-time point.

The experiment shown in Fig. 5g was performed three times independently, and each blot was used for the quantification shown in Extended Data Fig. 7f. The experiments shown in Figs. 2c–e, 3c,d and 5a–c,e-f and Extended Data Figs. 3b and 7a,b,d,e were performed twice on separate days with similar results. The experiments shown in Figs. 3e,f, 4c–e and 5h and Extended Data Figs. 1a–c, 2c,d,f,g and 6d were performed once.

Statistical analyses were performed using GraphPad PRISM version 10. Data distribution was assumed to be normal, but this was not formally tested. No statistical method was used to predetermine sample size. No data were excluded from the analyses. The experiments were not randomized. The investigators were not blinded to allocation during experiments or outcome assessments.

### Reporting summary

Further information on research design is available in the Nature Portfolio Reporting Summary linked to this article.

## Online content

Any methods, additional references, Nature Portfolio reporting summaries, source data, extended data, supplementary information, acknowledgements, peer review information; details of author contributions and competing interests; and statements of data and code availability are available at 10.1038/s41594-025-01610-9.

## Supplementary information

Reporting Summary

Supplementary Table 1 The spreadsheets for the results of the genome-wide CRISPR–Cas9 screen, proteomic analysis and crosslinking mass spectrometry.

## Source data

Source Data Fig. 2 Unprocessed western blots and/or gels.

Source Data Fig. 2 Statistical source data.

Source Data Fig. 3 Unprocessed western blots and/or gels.

Source Data Fig. 4 Unprocessed western blots and/or gels.

Source Data Fig. 4 Statistical source data.

Source Data Fig. 5 Unprocessed western blots and/or gels.

Source Data Extended Data Fig. 1 Unprocessed western blots and/or gels.

Source Data Extended Data Fig. 2 Unprocessed western blots and/or gels.

Source Data Extended Data Fig. 3 Unprocessed western blots and/or gels.

Source Data Extended Data Fig. 6 Unprocessed western blots and/or gels.

Source Data Extended Data Fig. 7 Unprocessed western blots and/or gels.

Source Data Extended Data Fig. 7 Statistical source data.

## Data Availability

Raw sequencing data and AlphaFold3 models that support the findings of this study were deposited to Mendeley data and were made available at 10.17632/85b999zcdz.1 (ref. ^79^). The mass spectrometry proteomics data have been deposited to the ProteomeXchange Consortium via the PRIDE partner repository with the dataset identifier PXD064067. Protein sequences were extracted from UniProt. All other data supporting the findings of this study are available from the corresponding authors on reasonable request. Source data are provided with this paper.
