# Supplementary material for: A heterotrimeric protein complex assembles the metazoan V-ATPase upon dissipation of proton gradients
Source: Nat Struct Mol Biol. 2025 Jul 11;32(10):2076–87. doi: 10.1038/s41594-025-01610-9 (PMC12527914; doi:10.1038/s41594-025-01610-9)
Supplement: Supplementary file 1 — Reporting Summary [file 41594_2025_1610_MOESM1_ESM.pdf]

Corresponding author(s): Christopher Nardone and Tom Rapoport

Last updated by author(s): 06/26/2025

Reporting Summary

Nature Portfolio wishes to improve the reproducibility of the work that we publish. This form provides structure for consistency and transparency in reporting. For further information on Nature Portfolio policies, see our Editorial Policies and the Editorial Policy Checklist.

Statistics

For all statistical analyses, confirm that the following items are present in the figure legend, table legend, main text, or Methods section.

n/a	Confirmed	
☐	☒	The exact sample size (*n*) for each experimental group/condition, given as a discrete number and unit of measurement
☐	☒	A statement on whether measurements were taken from distinct samples or whether the same sample was measured repeatedly
☐	☒	The statistical test(s) used AND whether they are one- or two-sided<br>*Only common tests should be described solely by name; describe more complex techniques in the Methods section.*
☒	☐	A description of all covariates tested
☐	☒	A description of any assumptions or corrections, such as tests of normality and adjustment for multiple comparisons
☐	☒	A full description of the statistical parameters including central tendency (e.g. means) or other basic estimates (e.g. regression coefficient) AND variation (e.g. standard deviation) or associated estimates of uncertainty (e.g. confidence intervals)
☐	☒	For null hypothesis testing, the test statistic (e.g. *F*, *t*, *r*) with confidence intervals, effect sizes, degrees of freedom and *P* value noted<br>*Give P values as exact values whenever suitable.*
☒	☐	For Bayesian analysis, information on the choice of priors and Markov chain Monte Carlo settings
☒	☐	For hierarchical and complex designs, identification of the appropriate level for tests and full reporting of outcomes
☒	☐	Estimates of effect sizes (e.g. Cohen's *d*, Pearson's *r*), indicating how they were calculated

Our web collection on statistics for biologists contains articles on many of the points above.

Software and code

Policy information about availability of computer code

Data collection	CytExpertv2.6
Data analysis	FlowJo v10.7.1, ChimeraX-1.4 - 1.7, GraphPad Prism v10, RELION v4.0.1, ImageJ 1.54g

For manuscripts utilizing custom algorithms or software that are central to the research but not yet described in published literature, software must be made available to editors and reviewers. We strongly encourage code deposition in a community repository (e.g. GitHub). See the Nature Portfolio guidelines for submitting code & software for further information.

Data

Policy information about availability of data

All manuscripts must include a data availability statement. This statement should provide the following information, where applicable:

- Accession codes, unique identifiers, or web links for publicly available datasets
- A description of any restrictions on data availability
- For clinical datasets or third party data, please ensure that the statement adheres to our policy

Raw sequencing data and AlphaFold3 models that support the findings of this study were deposited to Mendeley data and were made available at https://doi.org/10.17632/85b999zcdz.1. The mass spectrometry proteomics data have been deposited to the ProteomeXchange Consortium via the PRIDE partner repository with the dataset identifier PXD064067. Protein sequences were extracted from UniProt. Source data have been provided in Source Data. All other data

supporting the findings of this study are available from the corresponding author upon reasonable request: christopher_nardone@hms.harvard.edu and tom_rapoport@hms.harvard.edu.

Research involving human participants, their data, or biological material

Policy information about studies with human participants or human data. See also policy information about sex, gender (identity/presentation), and sexual orientation and race, ethnicity and racism.

Reporting on sex and gender	N/A
Reporting on race, ethnicity, or other socially relevant groupings	N/A
Population characteristics	N/A
Recruitment	N/A
Ethics oversight	N/A

Note that full information on the approval of the study protocol must also be provided in the manuscript.

Field-specific reporting

Please select the one below that is the best fit for your research. If you are not sure, read the appropriate sections before making your selection.

☒ Life sciences ☐ Behavioural & social sciences ☐ Ecological, evolutionary & environmental sciences

For a reference copy of the document with all sections, see nature.com/documents/nr-reporting-summary-flat.pdf

Life sciences study design

All studies must disclose on these points even when the disclosure is negative.

Sample size	Sample sizes were not predetermined.
Data exclusions	No data was excluded.
Replication	All key findings were reproduced at least twice independently.
Randomization	No group allocation was performed in any experiments.
Blinding	Blinding was not performed as subjective analysis was not needed and no group allocation was performed for any experiments

Reporting for specific materials, systems and methods

We require information from authors about some types of materials, experimental systems and methods used in many studies. Here, indicate whether each material, system or method listed is relevant to your study. If you are not sure if a list item applies to your research, read the appropriate section before selecting a response.

Materials & experimental systems

n/a	Involved in the study	
☐	☒	Antibodies
☐	☒	Eukaryotic cell lines
☒	☐	Palaeontology and archaeology
☒	☐	Animals and other organisms
☒	☐	Clinical data
☒	☐	Dual use research of concern
☒	☐	Plants

Methods

n/a	Involved in the study	
☒	☐	ChIP-seq
☐	☒	Flow cytometry
☒	☐	MRI-based neuroimaging

Antibodies

Antibodies used	All primary antibodies were diluted to 1:1000 by volume in the blocking solution and incubated overnight with gentle rocking at 4°C.

The following primary antibodies were used: rabbit anti-FLAG (Cell Signaling Technology, 14793, RRID:AB_2572291), anti-Actin (Cell Signaling Technology, 4970, RRID:AB_2223172), rabbit anti-HA (Cell Signaling Technology, 3724, RRID:AB_1549585), rabbit anti-mTOR (Cell Signaling Technology, 2983, RRID:AB_2105622), rabbit anti-ROGDI (Proteintech, 17047-1-AP, RRID:AB_2182328), rabbit anti-DMXL2 (Abcam, ab234771, RRID:AB_3675331), rabbit anti-ATP6V1A (Proteintech, 17115-1-AP, RRID:AB_2290195), rabbit anti-ATP6V1B2 (Cell Signaling Technology, 14617, RRID:AB_2798541), rabbit anti-ATP6V1C1 (Proteintech, 16054-1-AP, RRID:AB_2062501), rabbit anti-ATP6V1D (Proteintech, 14920-1-AP, RRID:AB_2243302), rabbit anti-ATP6V1E1 (Proteintech, 15280-1-AP, RRID:AB_2062545), rabbit anti-ATP6V1F (Proteintech, 17725-1-AP, RRID:AB_2062680), rabbit anti-ATP6V1G2 (Proteintech, 25316-1-AP, RRID:AB_2880027), rabbit anti-ATP6V1H (Proteintech, 26683-1-AP, RRID:AB_2880601), rabbit anti-ATP6V0A1 (Proteintech, 13828-1-AP, RRID:AB_2877979), rabbit anti-ATP6V0A2 (Abcam, ab96803, RRID:AB_10680914), rabbit anti-SKP1 (Cell Signaling Technology, 2156, RRID:AB_2270271), rabbit anti-ATG16L1 (Cell Signaling Technology, 8089, RRID:AB_10950320), rabbit anti-LC3 (Cosmo Bio, MBL-PM036, RRID:AB_2274121), rabbit anti-PSMD4 (CST, 12441, RRID:AB_2797916), and mouse anti-LAMP2 (Santa Cruz Biotechnology, sc-18822, RRID:AB_626858).

The following secondary antibodies were diluted 1:2000 by volume in blocking solution: anti-rabbit IgG, HRP-linked secondary antibody (Cell Signaling Technology, 7074, RRID:AB_2099233) or anti-mouse IgG, HRP-linked secondary antibody (Cell Signaling Technology, 7076, RRID:AB_330924).

The following magnetic beads were used for immunoprecipitations: anti-FLAG (Sigma, M8823, RRID: AB_2637089) or anti-HA (Thermo Fisher Scientific, 88836, RRID: AB_2749815).
For protein purification: M2 FLAG agarose resin (Millipore, A2220, RRID:AB_10063035)

Validation

The anti-V1 antibodies were independently validated during the course of the study, for example in figure 2d, using purified V1 protein. All other antibodies used in this study were used extensively in the field and authenticated by the provider.

Eukaryotic cell lines

Policy information about cell lines and Sex and Gender in Research

Cell line source(s)

Human embryonic kidney (HEK)-293T cells (ATCC, CRL-3216, RRID: CVCL_0063).

Authentication

Cell lines were not formally authenticated because they were obtained by a reputable provider. However, by imaging the cells using light microscopy, they exhibited the characteristic morphology of HEK-293T cells. The cells were indirectly authenticated also by their use in successfully packaging lentivirus.

Mycoplasma contamination

All cell lines were negative for mycoplasma as tested by staining and qPCR.

Commonly misidentified lines
(See ICLAC register)

None.

Plants

Seed stocks

Report on the source of all seed stocks or other plant material used. If applicable, state the seed stock centre and catalogue number. If plant specimens were collected from the field, describe the collection location, date and sampling procedures.

Novel plant genotypes

Describe the methods by which all novel plant genotypes were produced. This includes those generated by transgenic approaches, gene editing, chemical/radiation-based mutagenesis and hybridization. For transgenic lines, describe the transformation method, the number of independent lines analyzed and the generation upon which experiments were performed. For gene-edited lines, describe the editor used, the endogenous sequence targeted for editing, the targeting guide RNA sequence (if applicable) and how the editor was applied.

Authentication

Describe any authentication procedures for each seed stock used or novel genotype generated. Describe any experiments used to assess the effect of a mutation and, where applicable, how potential secondary effects (e.g. second site T-DNA insertions, mosiacism, off-target gene editing) were examined.

Flow Cytometry

Plots

Confirm that:

☒ The axis labels state the marker and fluorochrome used (e.g. CD4-FITC).

☒ The axis scales are clearly visible. Include numbers along axes only for bottom left plot of group (a 'group' is an analysis of identical markers).

☒ All plots are contour plots with outliers or pseudocolor plots.

☒ A numerical value for number of cells or percentage (with statistics) is provided.

Methodology

Sample preparation

HEK-293T cells were plated in 6-well plates and, two days later, pre-treated with 1 µM dTAG13 for 4 hours. The cells were

Sample preparation	then treated with 100 nM BafA1 for 1 hour in the presence of dTAG13. After treatment, the medium was discarded, and the cells were washed twice with fresh medium. After the second wash, fresh medium containing dTAG13 was added, and the cells were incubated for 2 hours at 37°C to recover lysosomal acidification. The medium was then removed, and the cells were stained with 1 µM LysoTracker (Thermo Fisher Scientific, L7528) for 1 hour at 37°C. Following the stain, the cells were rinsed once with PBS, detached using 0.05% trypsin, and the trypsin was quenched with fresh medium.<br><br>For neurotransmitter loading, HEK-293T cells were first infected with lentivirus to stably express VMAT2 and then selected with blasticidin. The same recovery procedure was followed, but instead of using LysoTracker, the cells were stained with 5 µM FFN206 (Tocris, 5043) for 2 hours. Approximately 10,000 individual cells were analyzed for YG602-A (LysoTracker) or V450-A (FFN206) using a CytoFLEX S flow cytometer. Flow cytometry data were collected using CytExpert software (Beckman Coulter), and figures were created using the FlowJo software.
Instrument	CytoFLEX S flow cytometer (Beckman Coulter, V2-B2-Y3-R2 version #C09762)
Software	CytExpert software (Beckman Coulter), FlowJo
Cell population abundance	The final YG602-A or V450-A positive cell population used for analysis were about 10,000 cells per condition.
Gating strategy	Gate1: FSC-A/SSC-A, Gate2: FSC-A/FSC-H, Gate3: YG602-A or V450-A.

☒ Tick this box to confirm that a figure exemplifying the gating strategy is provided in the Supplementary Information.
