# Supplementary figures and images for: A heterotrimeric protein complex assembles the metazoan V-ATPase upon dissipation of proton gradients
Source: Nat Struct Mol Biol. 2025 Jul 11;32(10):2076–87. doi: 10.1038/s41594-025-01610-9 (PMC12527914; doi:10.1038/s41594-025-01610-9)

Figure 2

Figure 2c

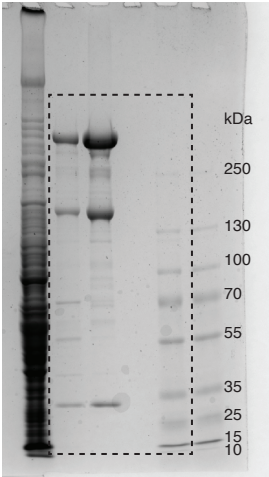

Figure 2e

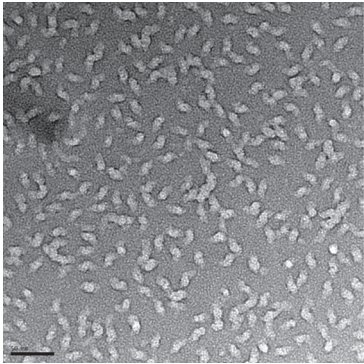

Figure 2d

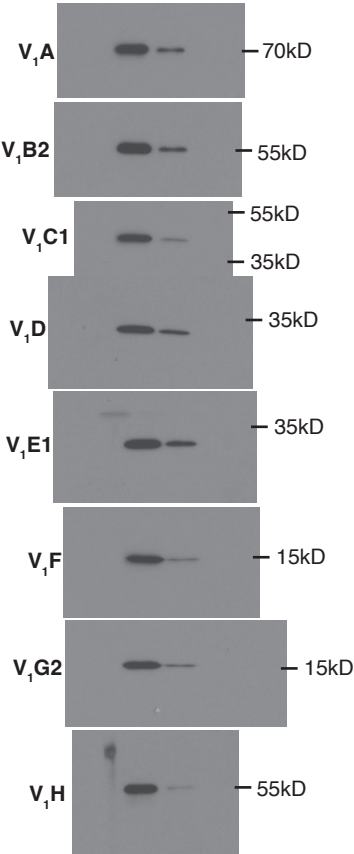

Supplement: Supplementary file 3 — Unprocessed western blots and/or gels. [file 41594_2025_1610_MOESM3_ESM.pdf]

Figure 3

Figure 3c

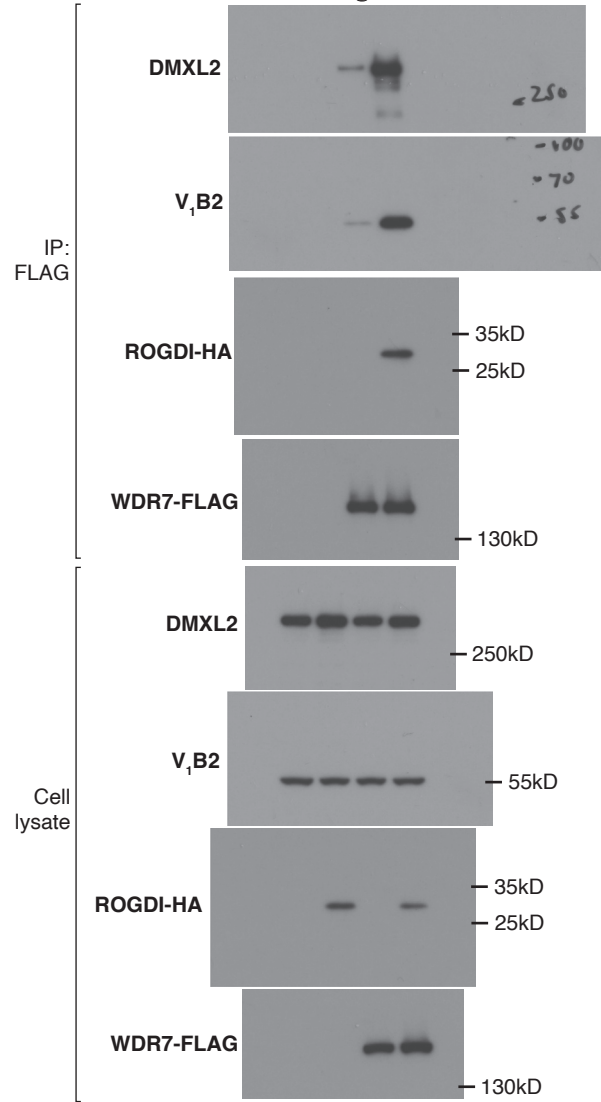

Figure 3d

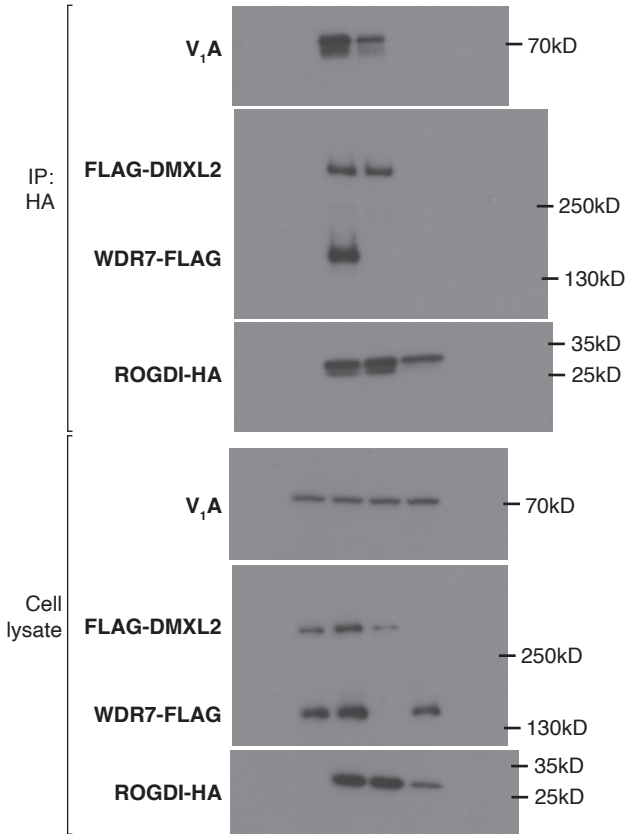

Figure 3

Figure 3e

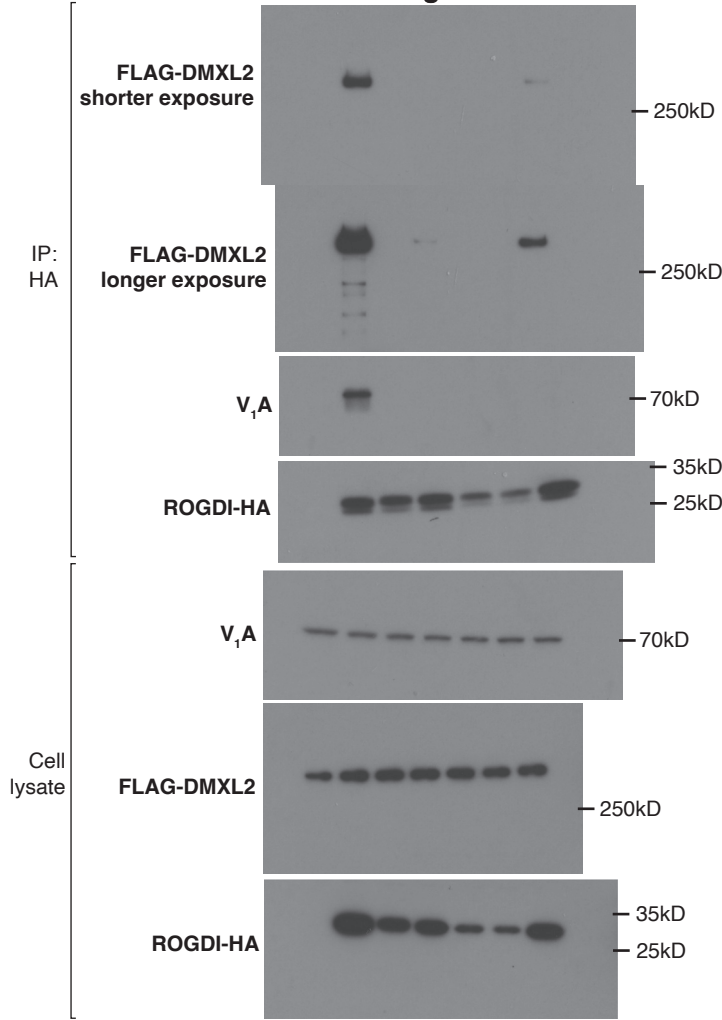

Figure 3f

IP: FLAG

Cell lysate

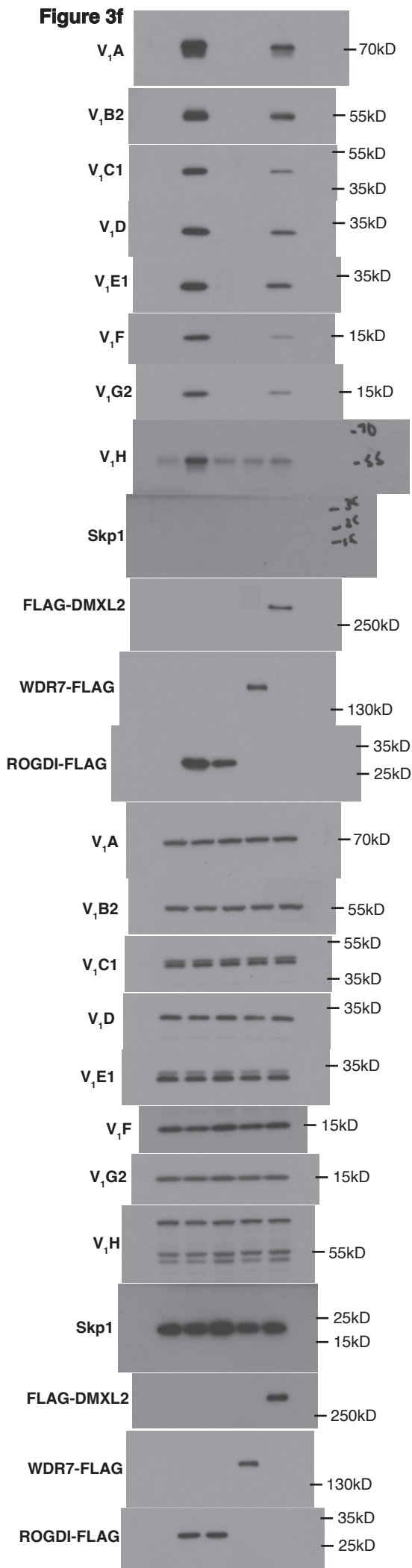

Supplement: Supplementary file 5 — Unprocessed western blots and/or gels. [file 41594_2025_1610_MOESM5_ESM.pdf]

**Figure 4**

**Figure 4c**

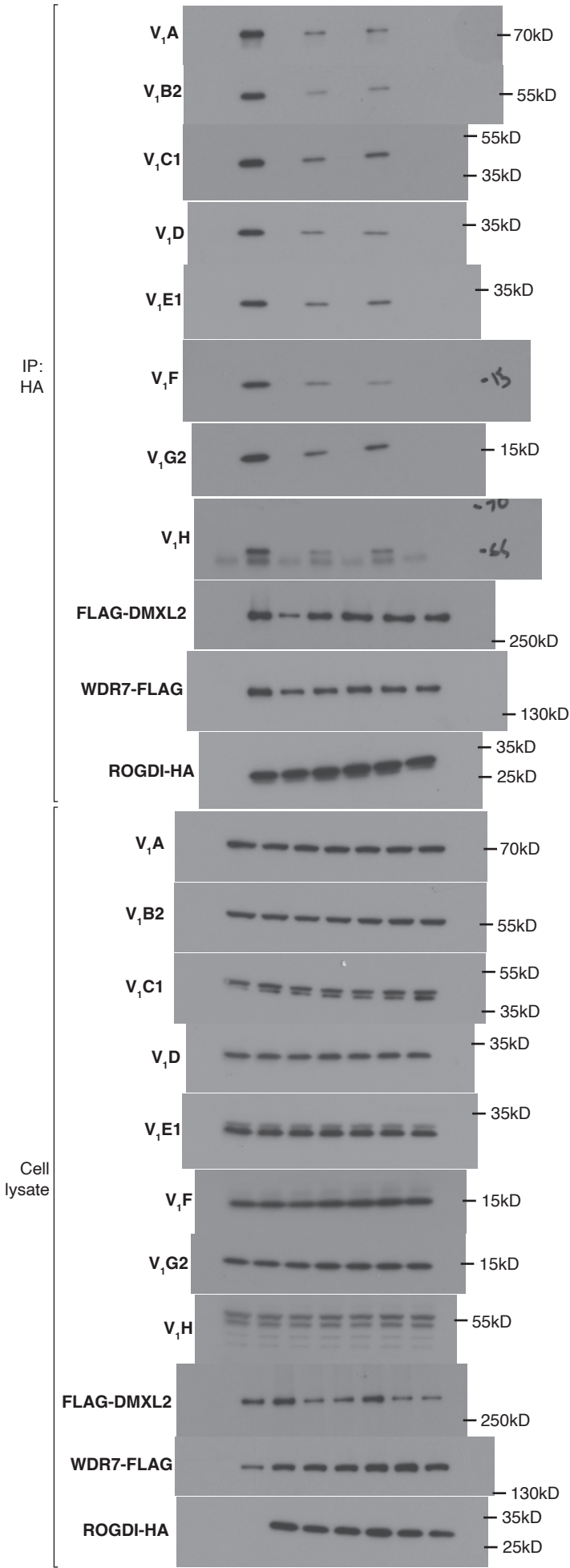

**Figure 4d**

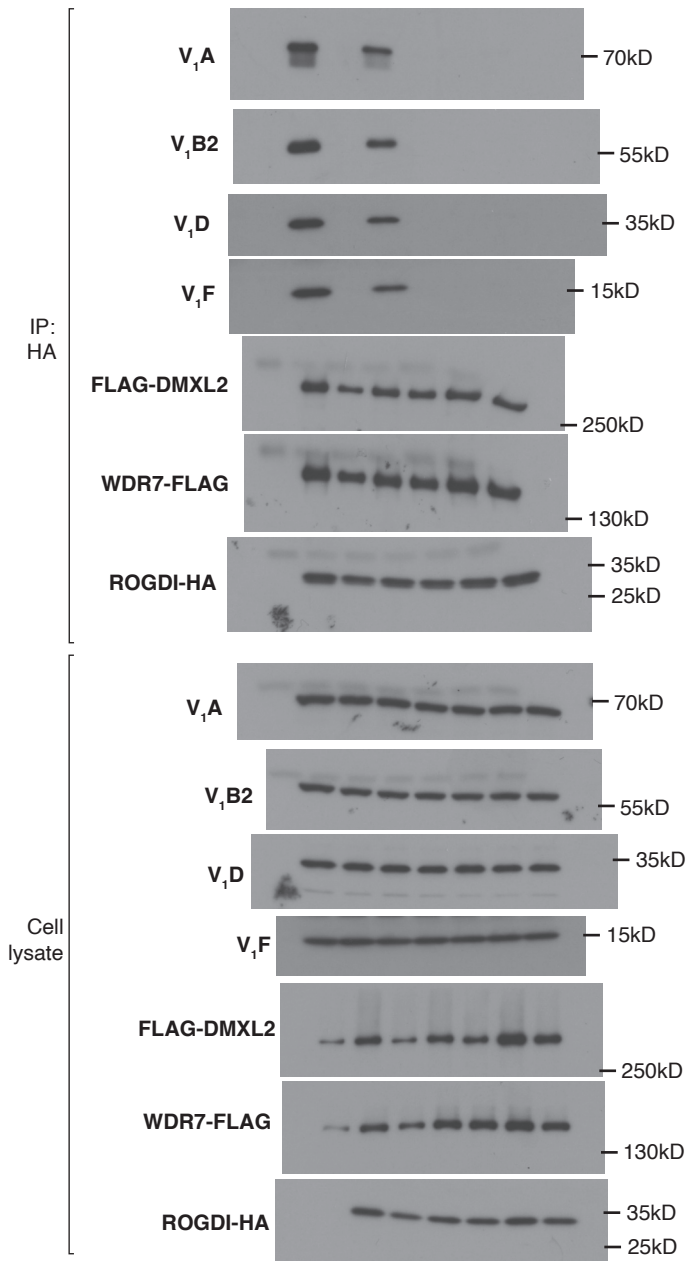

**Figure 4**

**Figure 4e**

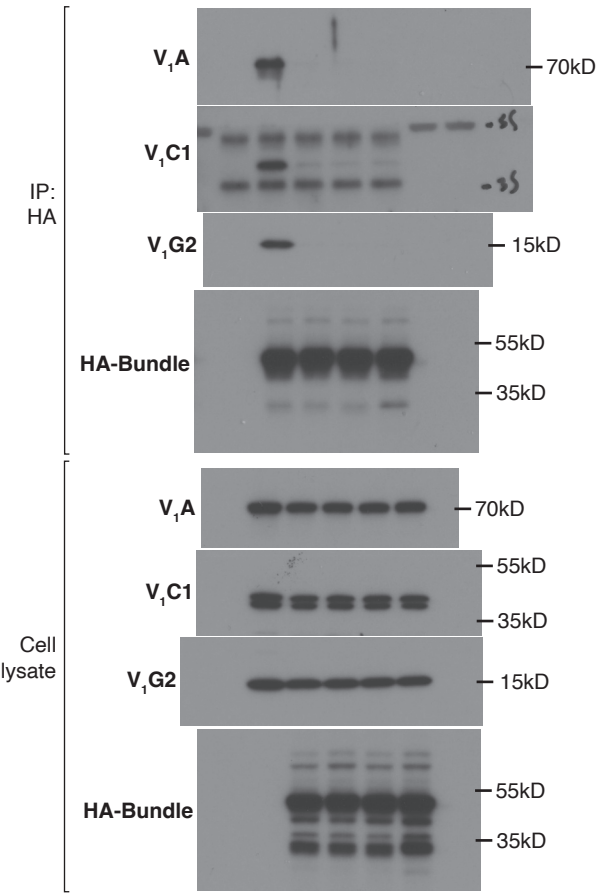

Supplement: Supplementary file 6 — Unprocessed western blots and/or gels. [file 41594_2025_1610_MOESM6_ESM.pdf]

Figure 5

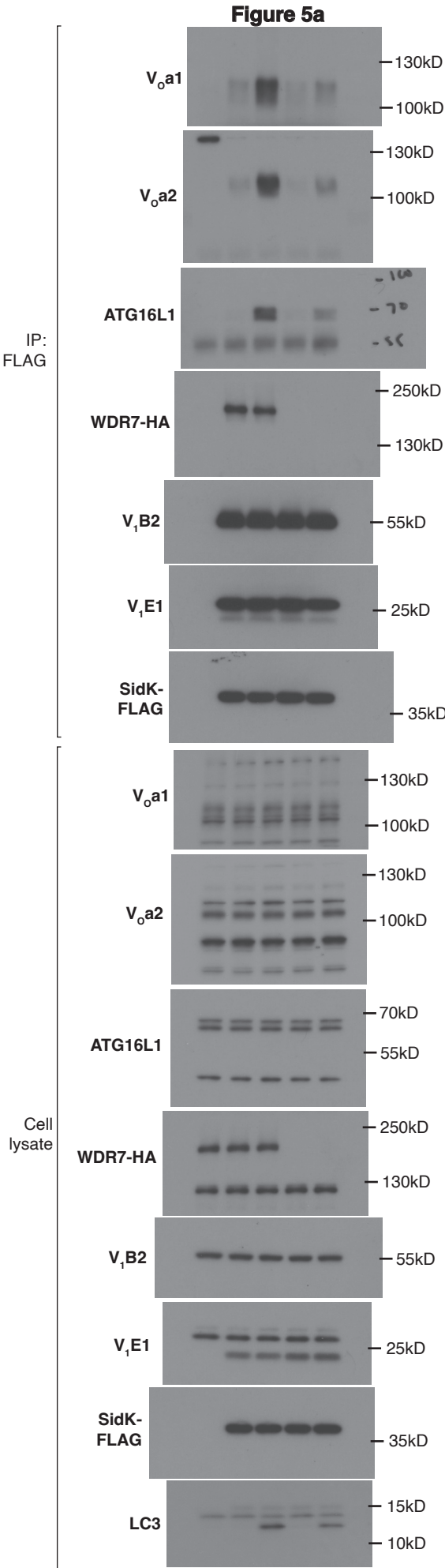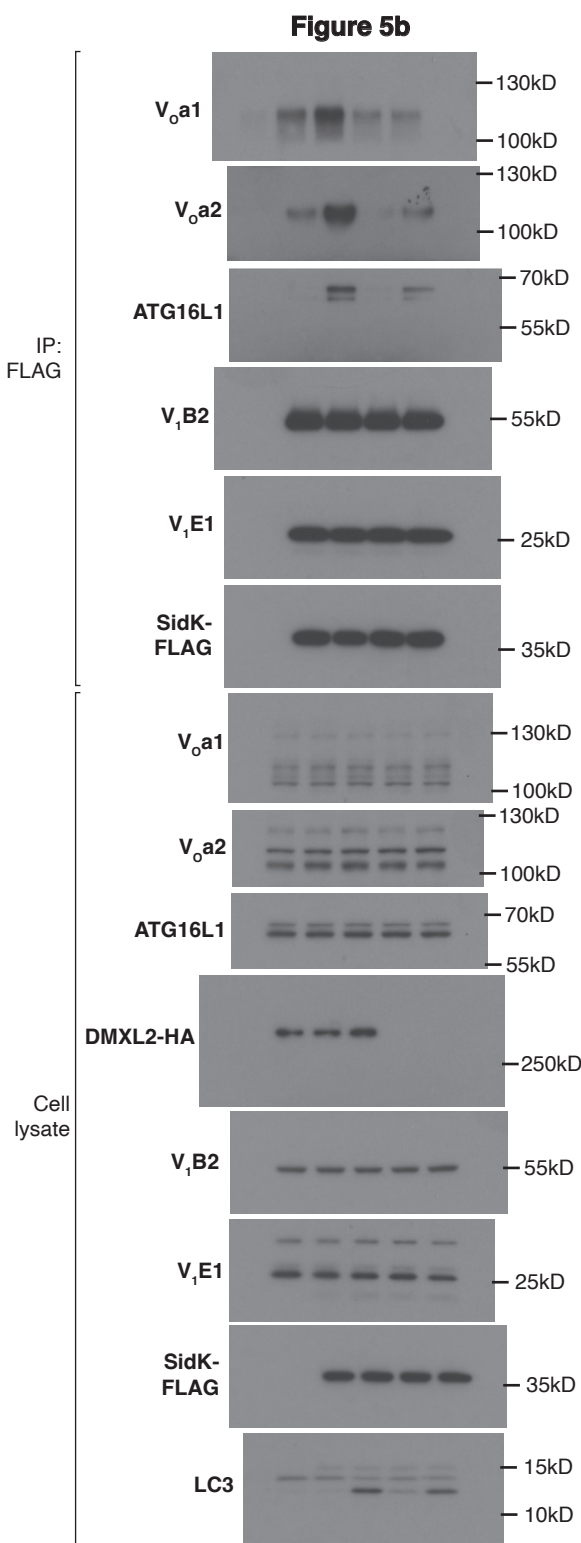

Figure 5

Figure 5c

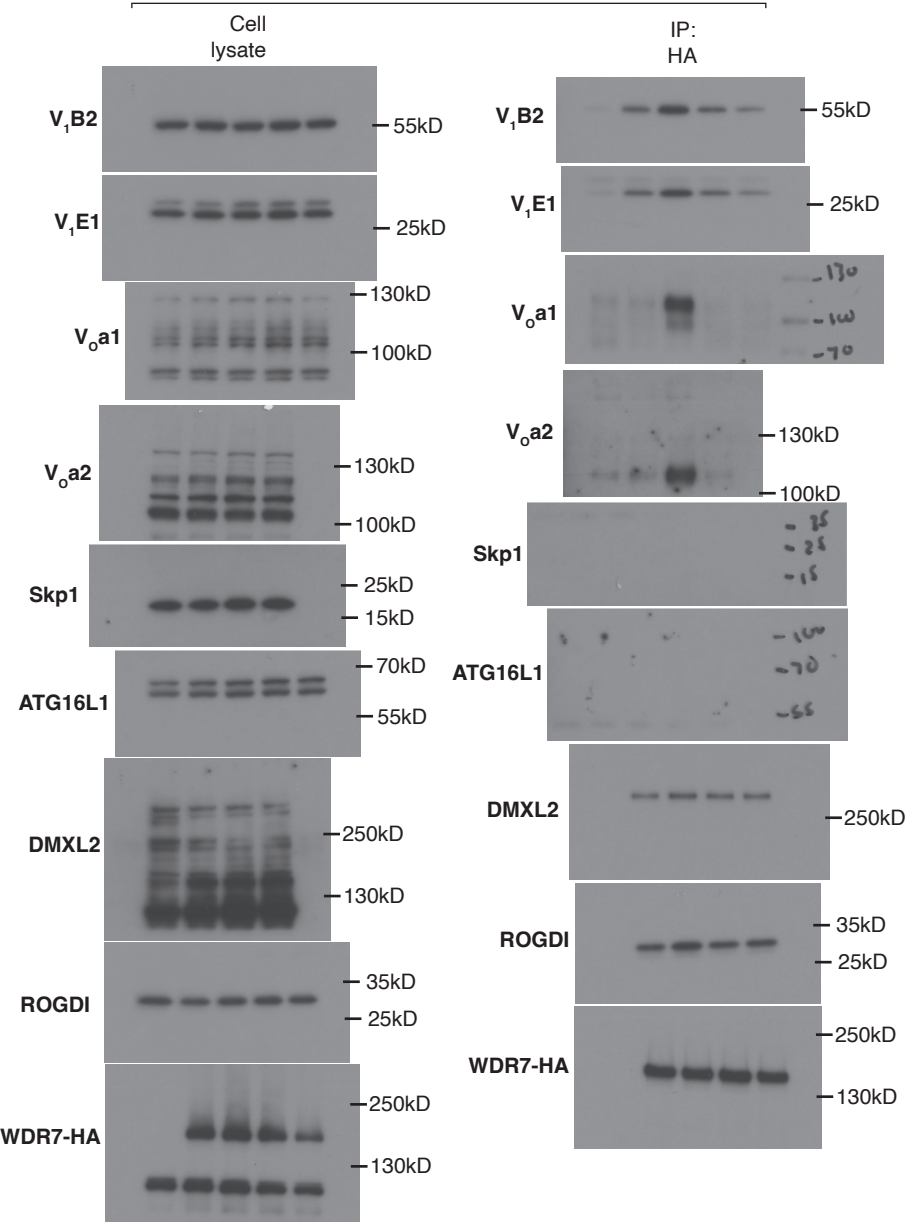

Figure 5e

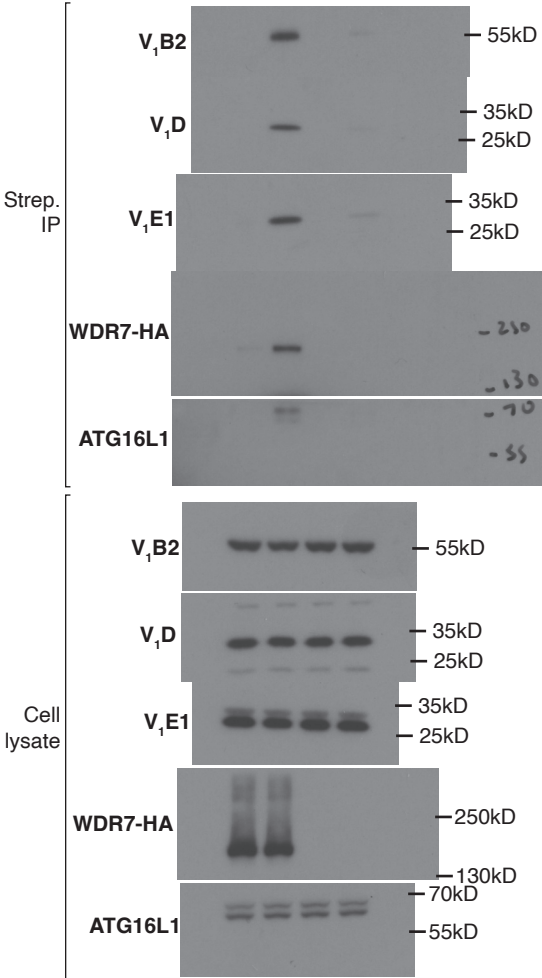

Figure 5f

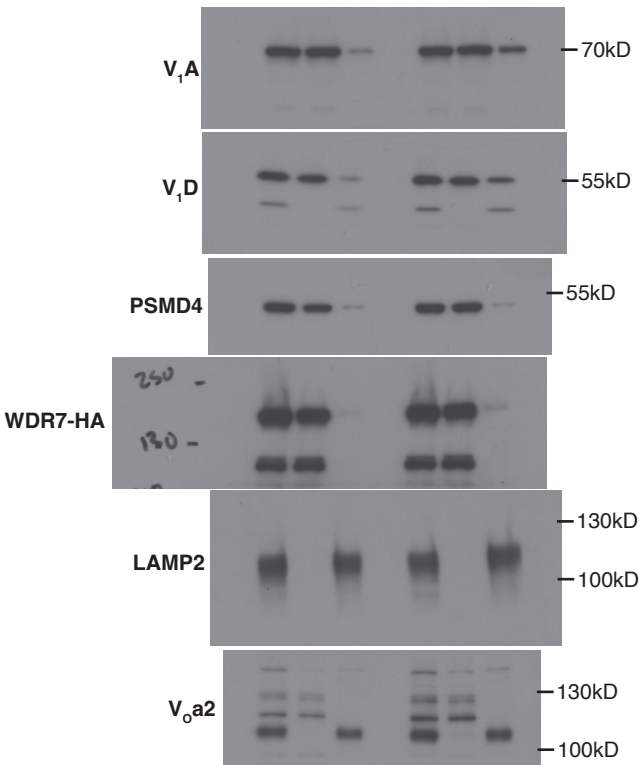

Figure 5

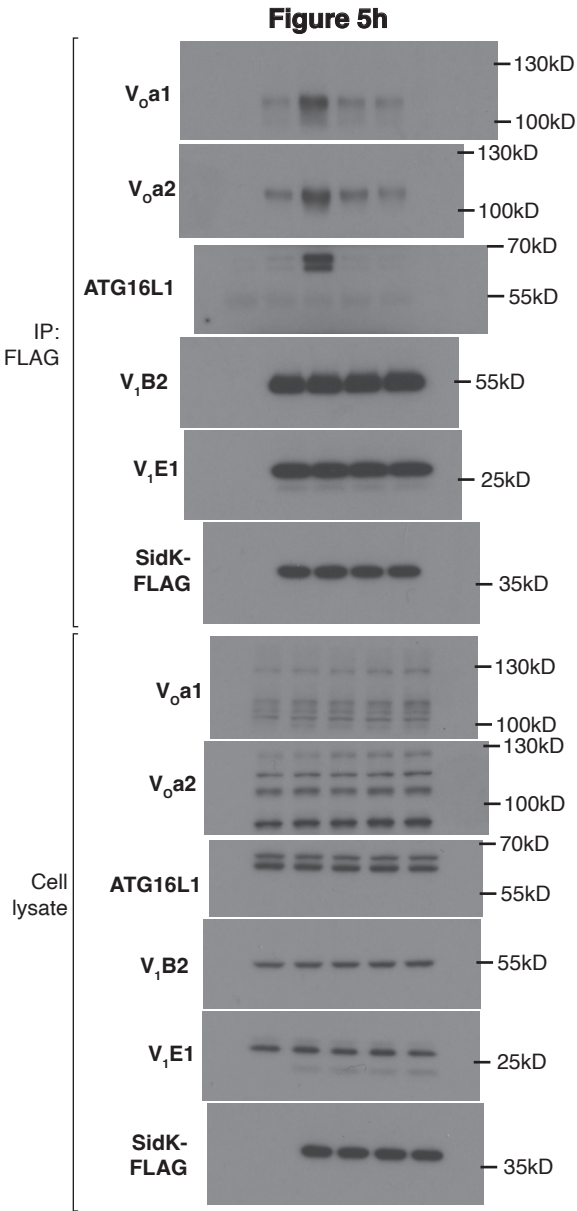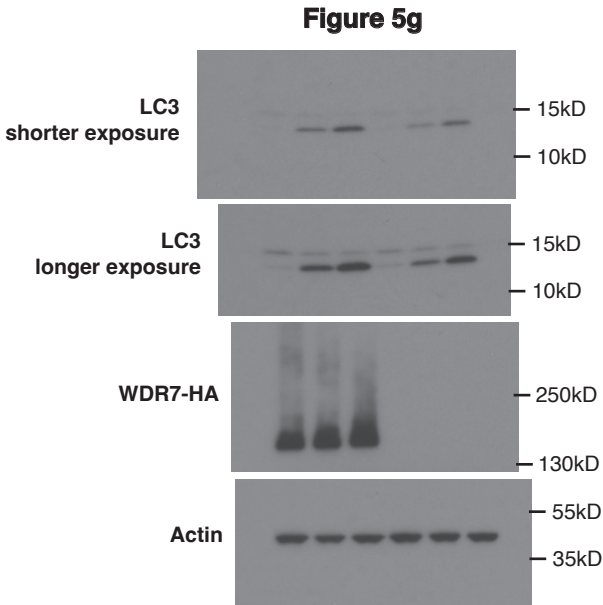

Supplement: Supplementary file 8 — Unprocessed western blots and/or gels. [file 41594_2025_1610_MOESM8_ESM.pdf]

Extended Figure 1

Ext Data Fig 1a

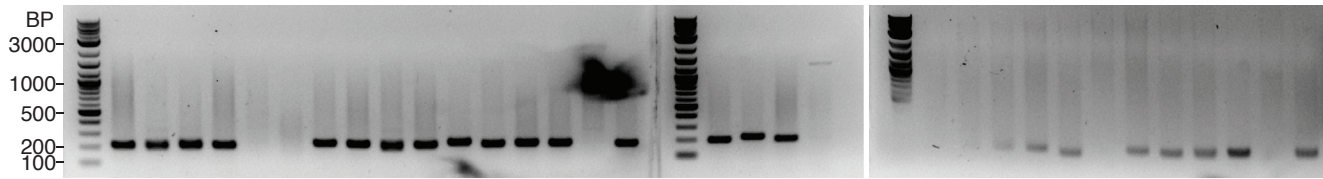

Ext Data Figure 1b

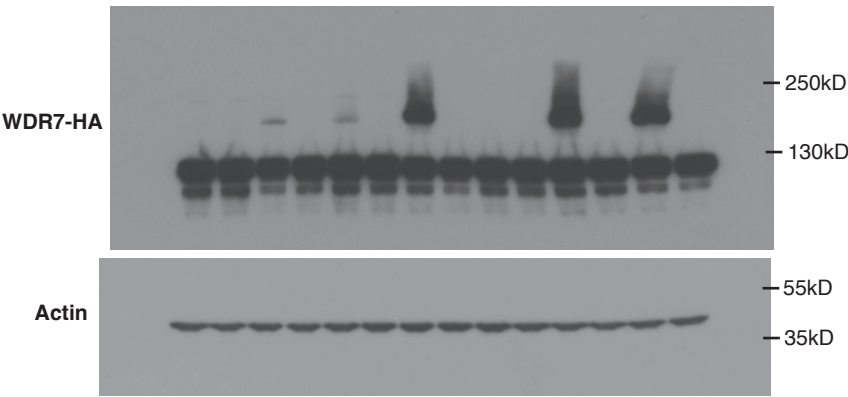

Ext Data Figure 1c

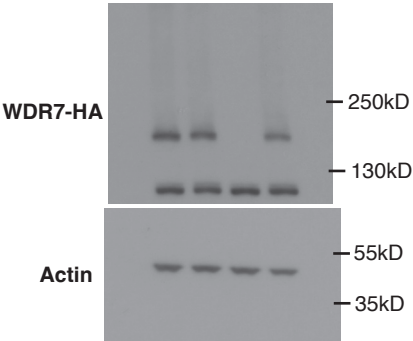

Supplement: Supplementary file 9 — Unprocessed western blots and/or gels. [file 41594_2025_1610_MOESM9_ESM.pdf]

**Extended Figure 2**

**Ext Data Fig 2c**

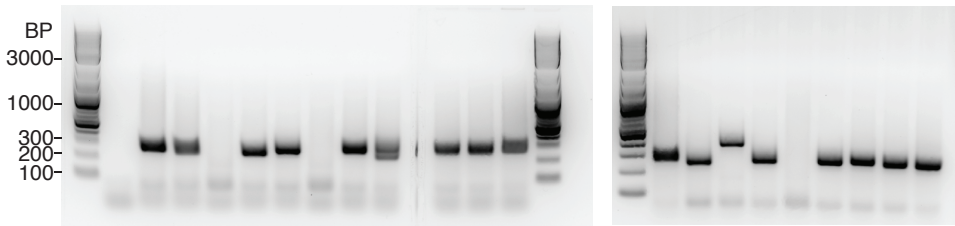

**Ext Data Figure 2d**

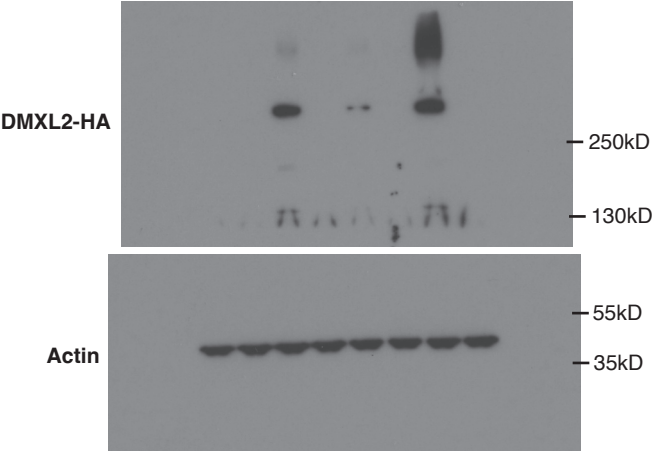

**Ext Data Figure 2f**

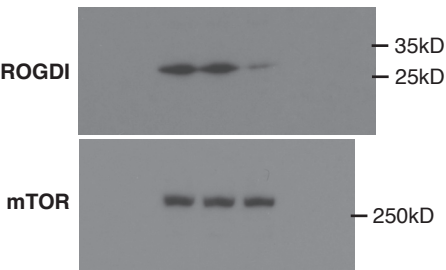

**Ext Data Figure 2g**

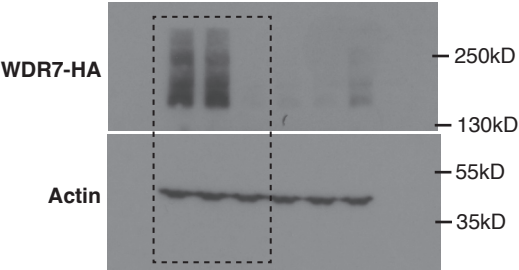

Supplement: Supplementary file 10 — Unprocessed western blots and/or gels. [file 41594_2025_1610_MOESM10_ESM.pdf]

Extended Figure 3

Ext Data Figure 3b

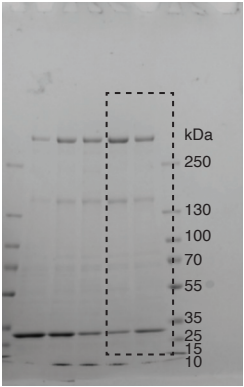

Supplement: Supplementary file 11 — Unprocessed western blots and/or gels. [file 41594_2025_1610_MOESM11_ESM.pdf]

Extended Figure 6

Ext Data Figure 6d

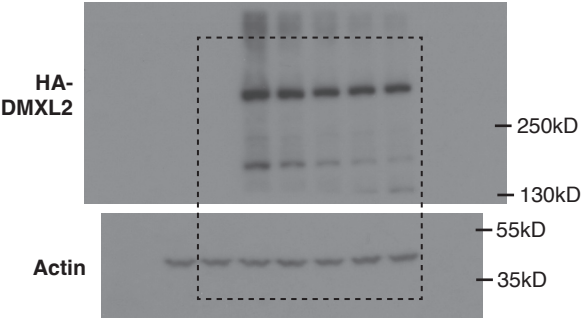

Supplement: Supplementary file 12 — Unprocessed western blots and/or gels. [file 41594_2025_1610_MOESM12_ESM.pdf]

Extended Figure 7

Ext Data Figure 7a

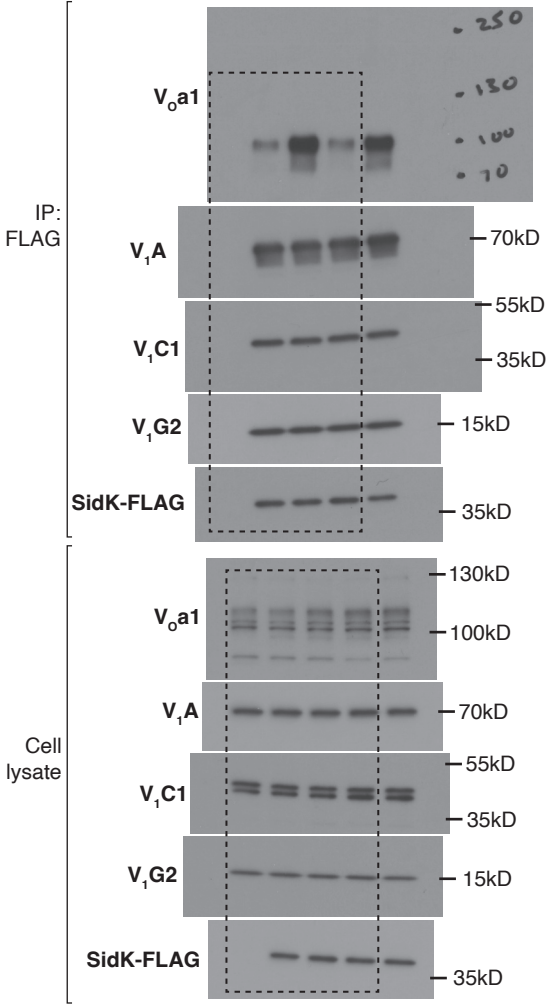

Ext Data Figure 7b

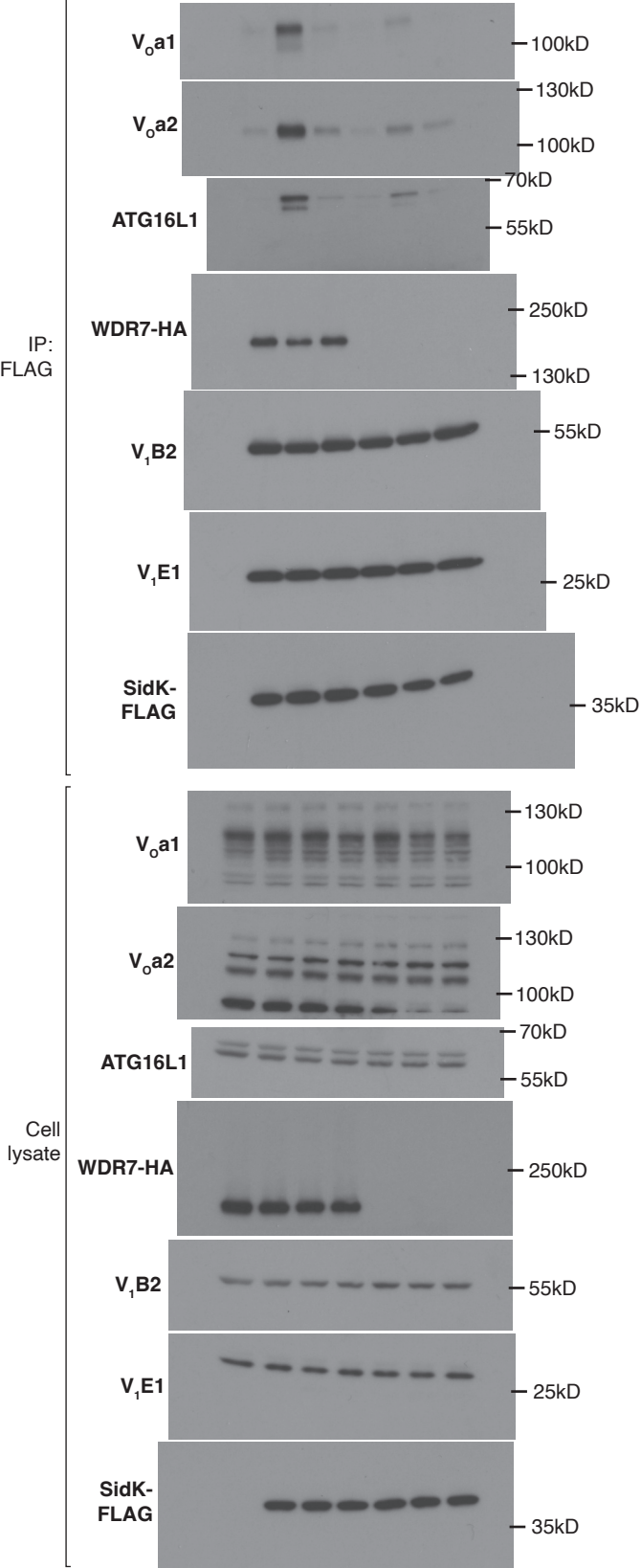

Extended Figure 7

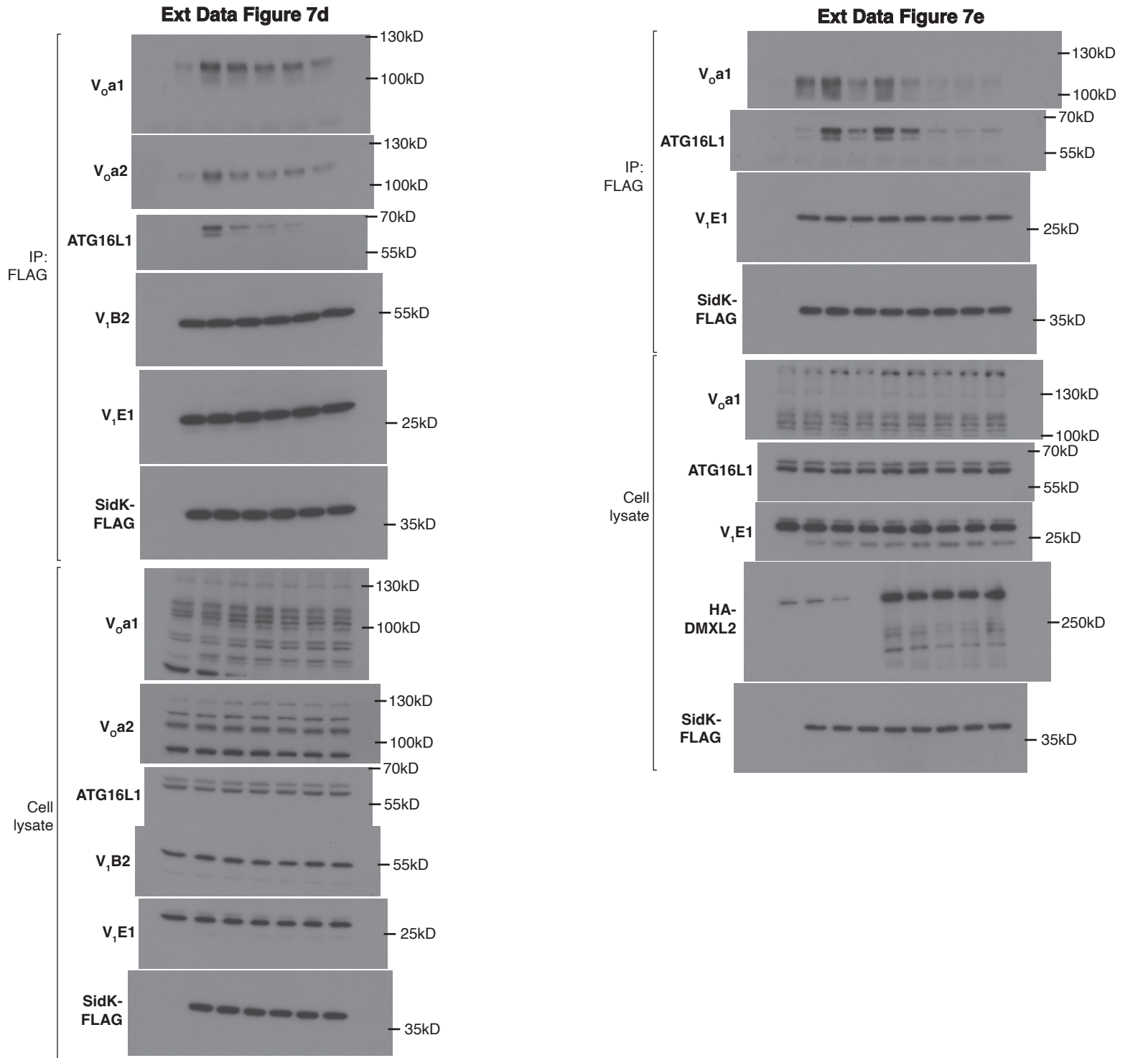

Supplement: Supplementary file 13 — Unprocessed western blots and/or gels. [file 41594_2025_1610_MOESM13_ESM.pdf]
